# A review on the recent applications of synthetic biopolymers in 3D printing for biomedical applications

**DOI:** 10.1007/s10856-023-06765-9

**Published:** 2023-11-20

**Authors:** Shiva S, Asuwin Prabu R G, Gauri Bajaj, Amy Elsa John, Sharan Chandran, Vishnu Vijay Kumar, Seeram Ramakrishna

**Affiliations:** 1 School of BioSciences and Technology, Department of Biotechnology, Vellore Institute of Technology, Vellore, Tamil Nadu 632014 India; 2 Centre for Nanotechnology and Sustainability, National University of Singapore, Singapore, 117575 Singapore; 3 School of Mechanical Engineering, Vellore Institute of Technology, Vellore, Tamil Nadu 632014 India; 4 Department of Ocean Engineering, Indian Institute of Technology Madras, Chennai, 600036 India; 5 Department of Mechanical and Industrial Engineering, Gadjah Mada University, Yogyakarta, 55281 Indonesia; 6 Department of Aerospace Engineering, Jain deemed to be University, Bangalore, India

## Abstract

3D printing technology is an emerging method that gained extensive attention from researchers worldwide, especially in the health and medical fields. Biopolymers are an emerging class of materials offering excellent properties and flexibility for additive manufacturing. Biopolymers are widely used in biomedical applications in biosensing, immunotherapy, drug delivery, tissue engineering and regeneration, implants, and medical devices. Various biodegradable and non-biodegradable polymeric materials are considered as bio-ink for 3d printing. Here, we offer an extensive literature review on the current applications of synthetic biopolymers in the field of 3D printing. A trend in the publication of biopolymers in the last 10 years are focused on the review by analyzing more than 100 publications. Their application and classification based on biodegradability are discussed. The various studies, along with their practical applications, are elaborated in the subsequent sections for polyethylene, polypropylene, polycaprolactone, polylactide, etc. for biomedical applications. The disadvantages of various biopolymers are discussed, and future perspectives like combating biocompatibility problems using 3D printed biomaterials to build compatible prosthetics are also discussed and the potential application of using resin with the combination of biopolymers to build customized implants, personalized drug delivery systems and organ on a chip technologies are expected to open a new set of chances for the development of healthcare and regenerative medicine in the future.

**Graphical Abstract:**

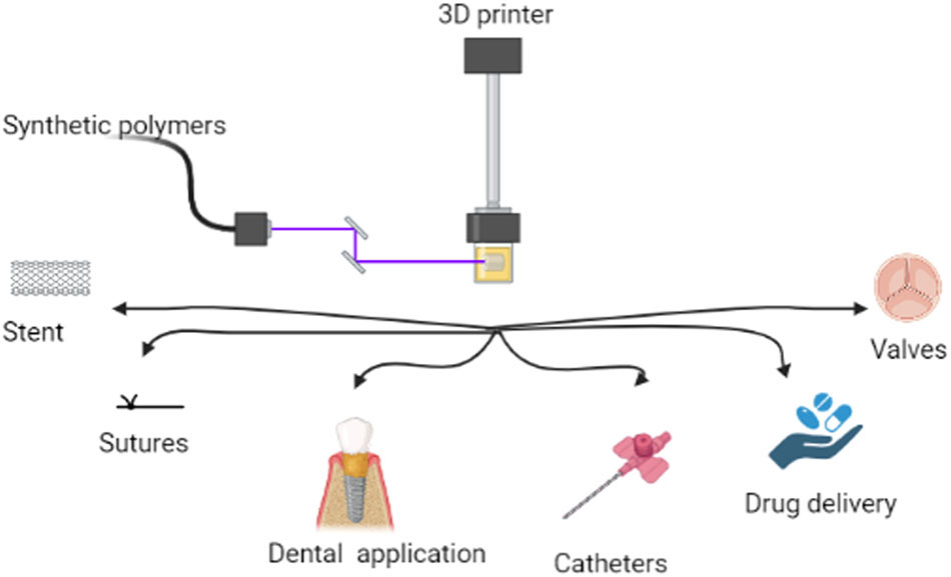

## Introduction

Long chains of monomeric components that are covalently bonded together to form polymeric biomolecules are called biopolymers [1]. Synthetic biopolymers are chemically synthesized from natural or synthetic monomers and designed to mimic the properties and functions of biopolymers found in nature. Due to their renewability, abundance, biodegradability, and other distinctive properties, including high adsorption capacity and ease of functionalization, they have been thoroughly explored for use in medical and other industrial applications [2]. Biomaterials are synthetically modified by functionalizing with hyaluronic acid derivatives or cell-instruction-labeled proteins for achieving biocompatibility and or target-specific applications [3].

In the food sector and medicinal research, natural biopolymers are used. The class of natural biopolymers includes collagen, chitin, fibrin, alginate, chitosan and hyaluronic acid, and many others. These materials are known to serve biomedical applications that provide therapeutic and lifesaving health benefits to humans [4]. Doctors, scientists, and bioengineers employ biomaterials for various medical implants, such as heart valves, stents, grafts, prosthetic joints, ligaments, tendons, implants for hearing loss, dental implants, and devices that stimulate neurons. Due to their biocompatibility, minimal immunogenicity, and prolonged duration, biomaterials have been made from natural polymers [5].

However, natural polymers pose several disadvantages such as poor mechanical properties and a high potential for contamination. The large-scale synthesis of natural polymers is another challenging task, and it is also difficult to change their properties [6]. Natural polymers can cause allergic reactions in some people since they are derived from biological sources. The best alternative for natural polymers is synthetic polymers. Synthetic polymers are less immunogenic than natural polymers and do not produce long-term immune-related inflammation [7]. Synthetic polymers outperform natural polymers in terms of mechanical properties [8, 9]. The synthetic polymers’ biodegradability can be altered, making them useful for tissue engineering and regenerative medicine. Synthetic polymers include including polylactic acid (PLA) [10], polyvinyl alcohol (PVA), polycaprolactone (PCL), and polylactic-co-glycolic acid (PLGA) [11] have attracted the greatest scientific interest. The chemical composition of various synthetic polymers is extensively studied to create biomaterials for various purposes [12].

Recent applications of synthetic polymers include tissue engineering and injectable drug delivery devices, which have evolved over the last 20 years [13]. Finding viable substitutes for deteriorating bodily components has been one of the main factors driving the use of polymers in medicine. The most common application of synthetic biopolymers so far has been in skeletal rebuilding [14]. Mechanical strength, ease of processing, biological inertness, abrasion resistance, wear-and-tear properties, blood compatibility, tissue adhesion, and oxygen permeability are a few parameters that impact polymer selection. The selection of biopolymer is often based on the chemical bonding between the monomeric unit which affects the mechanical strength and properties [15].

**Fig. 1:**
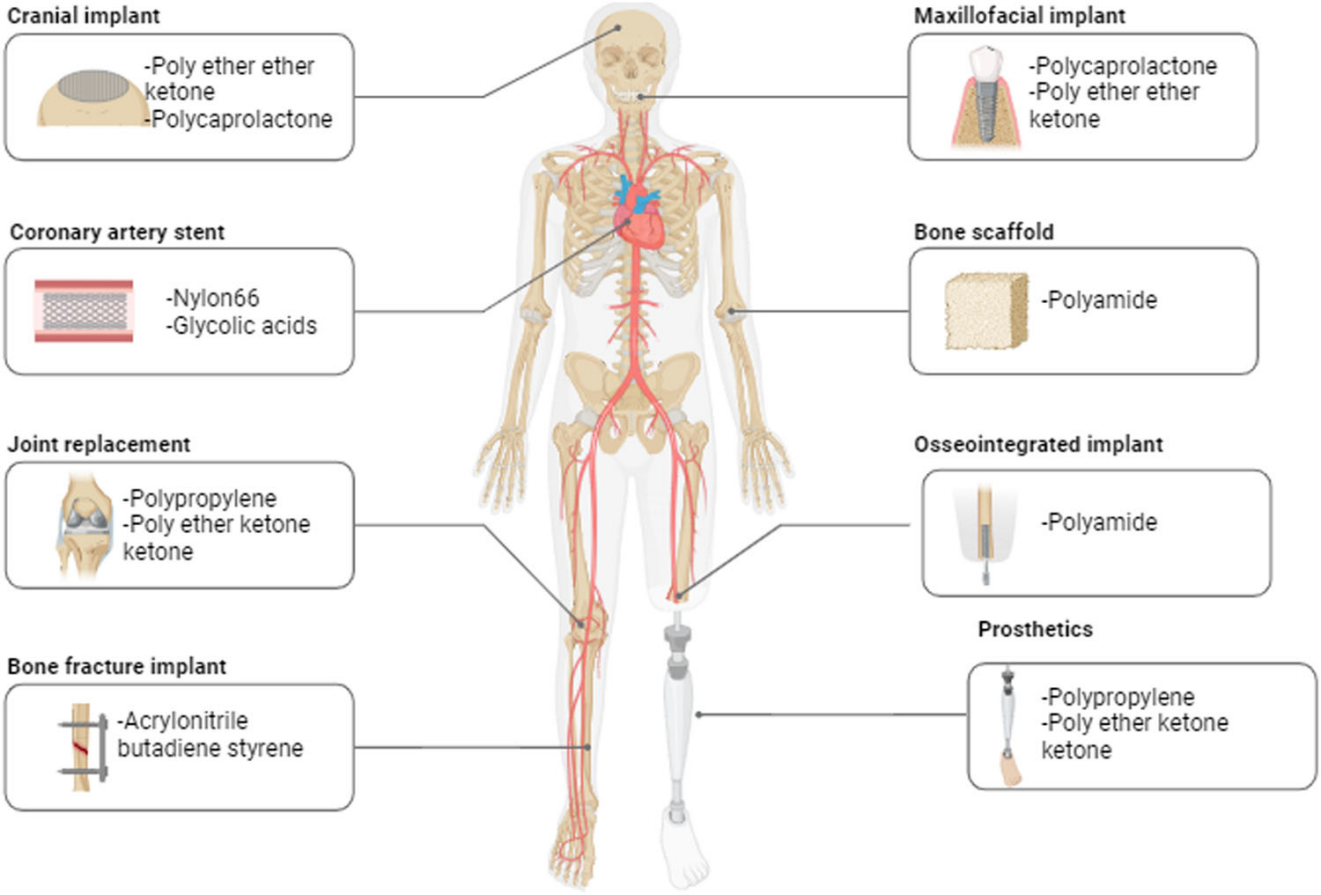
Applications of 3D printed synthetic polymers in area of osteology

Three-dimensional (3D) printing technology uses biopolymers to produce biomedical components, frequently to mimic natural tissue properties [16]. 3D bioprinting, in general, can employ a layer-by-layer process to deposit bio-inks to generate tissue-like structures that are later utilized in many medical and tissue engineering applications [17]. 3D bioprinting encompasses a wide variety of bioprinting processes and biopolymers. Currently, bioprinting is utilized to create tissue, sutures and organ models to aid in medication and therapy research [18, 19]. Figure 1 shows the diverse application of how 3d printed synthetic polymer can be helpful for bone regeneration and in cases of requirement of support.

This review paper discusses the various synthetic biopolymers and their application in 3D printing field which are briefly discussed in the table below. The polymers discussed in this review are Polypropylene, Polycaprolactone, Polylactide, Nylon, Polyether Ketone, Glycolic Acid, Acrylonitrile Butadiene Styrene, Polycarbonates and Polyamide. The recent developments of these polymers are discussed with their applications. Each section finally concludes with the advantages and disadvantages of using these biopolymers. A future direction with conclusion is provided at the end. The review is highly relevant and significant with increasing research in the area of 3D printing for biomedical application.

## Synthetic biopolymers

**Fig. 2:**
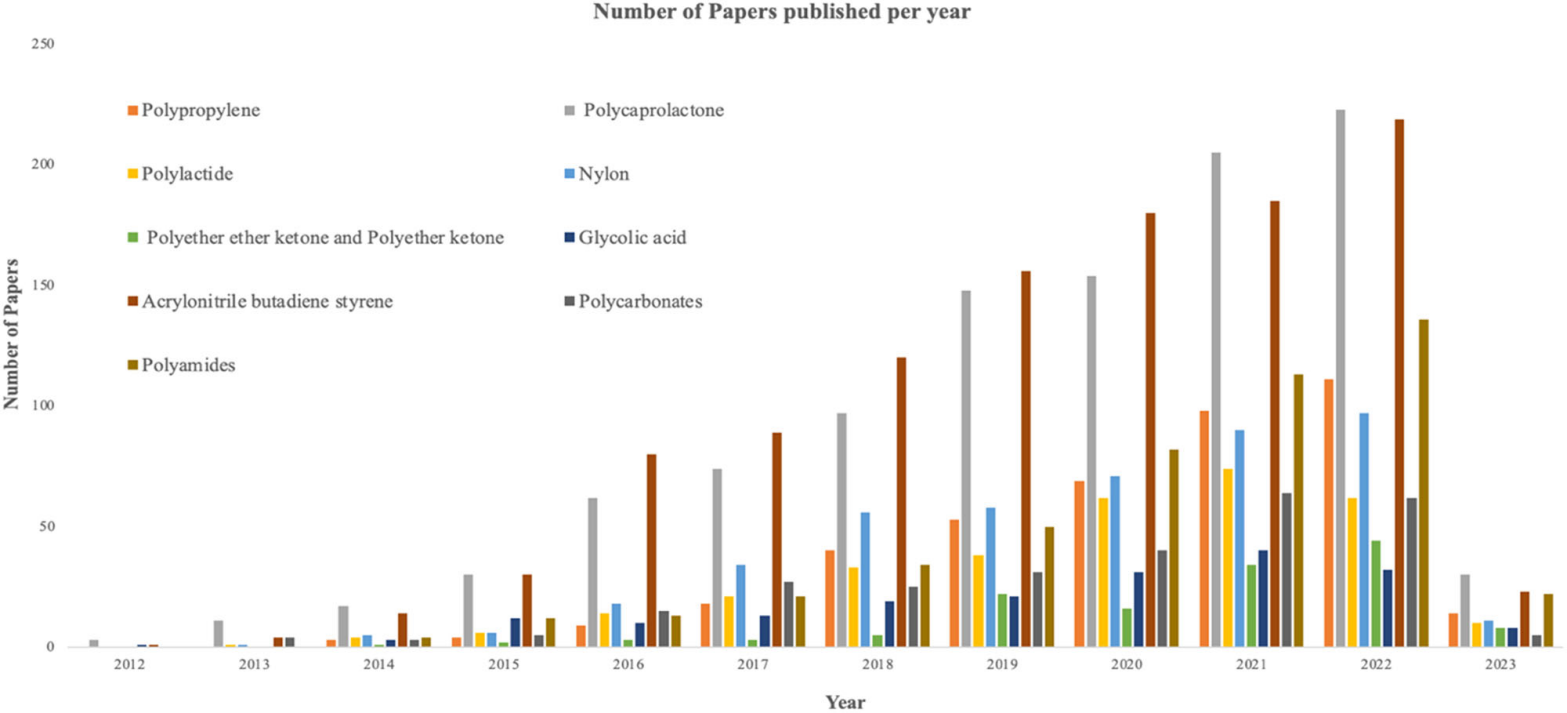
Number of publications per year in the field of 3D printing of biopolymers (Source: Web of Science, Keywords: “3D printing” + “ Polymer name”, from 2012 to 2023)

Figure 2 depicts the trend in the number of papers published from 2012 to 2023 for different synthetic biopolymers used in 3D printing, considered in this review. The trend keeps increasing rapidly showing the relevance of biopolymers employed in the 3D printing approach. The various synthetic biopolymers considered are given briefly in the coming sections.

### Polypropylene

Polypropylene has found various applications, including its use as sutures and prosthetics. The properties of PP are characterized by its molecular weight and dispersion, crystalline nature, and tacticity. It is a low-cost polymer with numerous properties such as flame resistance, transparency, dimensional stability, and recyclability [20, 21]. Polypropylene has low-strength applications due to its fatigue resistance and lightweight characteristics. It is semi-rigid and is majorly used to store and package (Tables 1 and 2). It has good heat and impact resistance and is transparent, due to which they are used in syringes, inhaling systems, containers, caps, and closures. Adding to this, it is low in density, has good chemical resistance, low toxicity, excellent electrical insulation, low moisture absorption, low flammability, and is easy to sterilize due to which it is used in pharmaceutical, medical devices, laboratories, syringes, and diagnostics fields [22]. The use of hemp fiber and coir fiber, combined with degradable and bioresorbable polypropylene, can create joints and bone fixtures that can relieve pain in patients and show a high elongation property [23]. Polypropylene also exhibits anti-thrombogenic, which can be enhanced to assist the adsorption of plasma proteins and its adhesion and activation of the platelet, which are very crucial for biomedical applications. The improvement seeks to increase blood compatibility by adding functional groups to the polymer’s surface using the cold atmospheric pressure plasma (CAPP) aided polymerization process [24].

**Table 1:** Applications of polymer and the chemical crosslink

Polymer	Applications	Structure
Polypropylene	• Sutures and prosthetics. • Syringes, inhaling systems, containers, caps, and closures. • Drug delivery.	
Polycaprolactone	• Scaffolds. • Drug delivery systems. • Bone healing.	
Polylactide	• Use in tissue engineering application. • Treatment of segmental bone defects. • It can be found in packaging, textiles, disposable cutlery, medical equipment such as cardiovascular implants and orthopedic devices.	
Nylon	• Wound closure methodologies. • Catheter balloons in treatment of cardiovascular disorders. • Nasopharyngeal swab.	
Polyether ether ketone	• Orthopedic implants such as hip joints, replacements for broken bones, and scaffolding. • Application in bone tissue engineering and craniofacial engineering.	
Glycolic acid	• Pharmaceutical applications. • Its use in load-bearing applications such as bone implants.	
Acrylonitrile butadiene styrene	• Its use in load-bearing applications, such as bone implants. • Medical masks. • Valves for ventilators.	
Polycarbonates	• Drug-delivery devices, from nebulizers to dialysis machines to needle-less safety syringes. • Provides a safety barrier that helps protect infants in incubators and allows medical personnel to monitor their health.	
Polyamide	• They are used in load-bearing applications, such as bone implants. • They are used for catheters that require stability and high precision tolerance at all atmospheric moisture content levels.

**Table 2:** Comparative properties of 3D printable polymers inclusive to synthetic polymers discussed in this paper

Polymer	Molecular weight	Modulus of elasticity	Hardness	Yield strength	Melting point	Friction coefficient	Tensile strength	Density	Elongation	Tg
Polyethylene	28.05 g/mol	1.08 GPa	7.75–9.71 Pa	23–29.5 MPa	110–140 °C	0.0270–0.300	7.60–43 MPa	0.9–1 g/cm^3^	11–13%	−80 °C
Poly propylene	354.6 g/mol	1.14–1.55 GPa	50 Pa	31–45 MPa	160–163 °C	0.23	31–45 MPa	0.905 g/cm^3^	11%	−25 °C
Poly caprolactone	114.4 g/mol	343.9–364.3 MPa	–	8.2–10.1 MPa	60 °C	–	10.5–16.1 MPa	1.1 g/cm^3^	–	−60 °C
Poly carbonates	272.29 g/mol	3.24 GPa	90 Pa	63 MPa	310 °C	0.31	66 MPa	1.23 g/cm^3^	1.30%	140–155 °C
Poly lactide	72.06 g/mol	2.4 GPa	–	26.082 MPa	170 °C	0.5	70 MPa	1.25 g/l	3.00%	60 °C
Poly amide	224.30 g/mol	2.93 GPa	70 Pa	40–100 Mpa	255 °C	0.114	90 MPa	1.14 g/cm^3^	0.2%	35–45 °C
Nylon 66	224.2994 g/mol	2.7 GPa	–		240–265 °C	0.1–1.5	25–90 MPa	1.14 g/cm³	60%	50–60 °C
Acronitrile butadiene styrene	211.30 g/mol	1.9–2.5 GPa	68–118 Pa	44.36 MPa	–	0.35	22–74 MPa	1115 kg/m^3^	–	105 °C

**Fig. 3:**
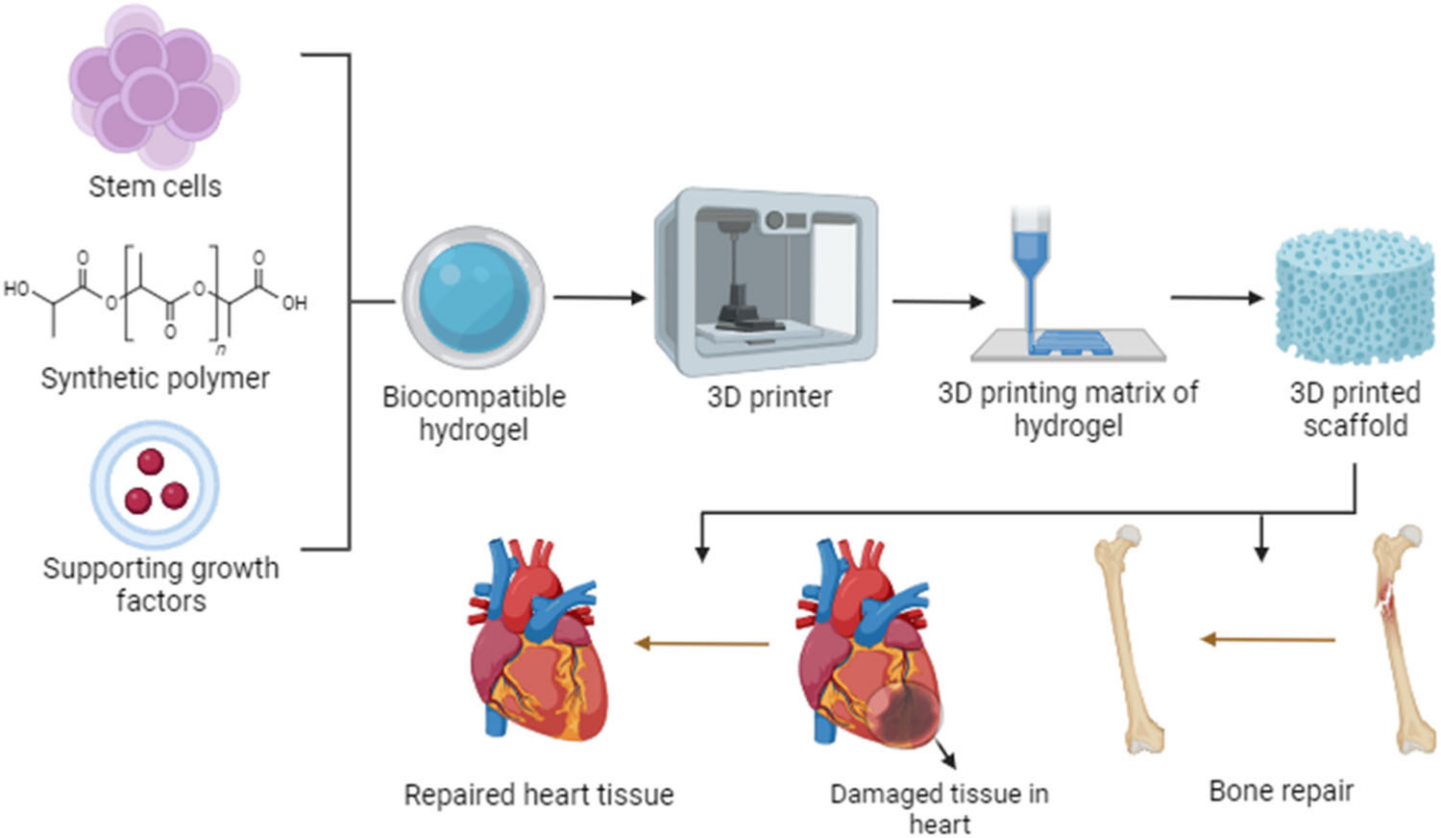
3D printing of synthetic polymer allowing stem cells to aid in bone repair and repair of damaged tissues

Biocompatibility of conventional sutures is always an alarming concern to immunological reactions, which is often called hypersensitivity arising from sutures where sutures act as an antigen and trigger an immune response. Polypropylene has been found as the perfect candidate to alleviate biocompatibility issues and provide a better therapeutic approach. The immune incompatibility affects the dermal layer irritation and concerns deep tissue and wound closure. Any such cases with incompatibility can therefore affect healthcare management and increase the healthcare cost; therefore, it should be consulted well before taking a specific suture [25]. Tissue rejection and erosion of the prosthetic mesh in the treatment of female pelvic floor dysfunction remained, for which the conventional treatment is by pelvic reconstruction using synthetic graft [26, 27]. The application of using polypropylene-based mesh incorporated with adipose-derived stem cells has found an application in the treatment of dysfunction with the knowledge that ADSCs have the ability of self-renewal and high differentiation potential, which facilitates tissue engineering [28–30]. The incorporation of stem cells in 3D printed scaffold is also graphically illustrated in Fig. 3.The inadequate mechanical properties of PP prevent it from being employed in load-bearing applications, however, due to its exceptional fiber-forming capabilities, it has been used to cure ventral incisional hernias and for supplying tetracycline in dental therapy. Research and development are still being done on chemical crosslinking of polypropylene for use in biomedical applications. There are certain potential strategies and factors to take into account to achieve the desired properties and biocompatibility. Utilizing functional groups or reactive molecules that can undergo crosslinking reactions is one approach that might be used. For instance, by adding thiol groups to polypropylene chains, crosslinking can be accomplished via thiol-ene or thiol-epoxy processes.

Advantages:

- Biocompatibility: polypropylene is thought to be biocompatible, which means that it is well accepted by humans and has no hazardous or severe side effects. Because of this, it can be utilized in a variety of biomedical applications, including as implants and medical equipment.
- Resistivity: acids, bases, and organic solvents are only a few of the many substances to which PP has good resistance. In biological applications where the material may interact with various substances or go through sterilizing procedures, this characteristic is advantageous.
- Mechanical characteristics: polypropylene has strong tensile characteristics, is rigid, and is impact resistant. Due of these characteristics, it can be used for structural integrity and durability-required applications like orthopedic implants and prosthetics.
- Cost-effectiveness: when compared to other polymers utilized in biomedical applications, like polyethylene and polycarbonate, polypropylene is a relatively cheap substance. This makes it an economical option for manufacturers of medical devices.
- Compatibility with sterilization: polypropylene is resistant to ethylene oxide, gamma radiation, and a number of other sterilizing techniques. This enables the sterilization of medical devices made of polypropylene without affecting their characteristics.

Disadvantages of using PP include:

- Poor resistance to UV radiation: polypropylene is not very resistant to UV radiation and can break down quickly when exposed to sunlight. This can make it unsuitable for outdoor applications where prolonged exposure to sunlight is expected.
- Low-temperature resistance: polypropylene has a relatively low melting point and can become brittle at low temperatures. This makes it unsuitable for use in applications that require exposure to extremely low temperatures.
- Flammable: polypropylene is a flammable material and can ignite easily. It also releases toxic fumes when burned, which can pose a risk to health and safety.
- Limited chemical resistance: although polypropylene is chemically resistant to a wide range of acids and bases, it can be affected by certain solvents and chemicals. It is important to ensure that it is compatible with the specific chemicals used in the application.
- Difficult to bond: polypropylene is difficult to bond using traditional adhesive methods. Specialized bonding agents and techniques are required to achieve a strong bond between polypropylene and other materials.
- Not biodegradable: polypropylene is not biodegradable and can persist in the environment for a long time. This can pose a risk to wildlife and the environment.

### Polycaprolactone

Polycaprolactone is a polymer that is commonly used in tissue engineering applications with polyester with excellent biocompatibility and biodegradability, as well as appreciable rheological characteristics, is polycaprolactone (PCL). It can stay stable for many days within its melting processing temperature range, but when temperatures reach 170 °C, it degrades considerably and fast. Additionally, it is perfect for melt operations because of its low melting point, changeable viscosity, and general adaptability. At 50 °C, a thermotolerant Aspergillus species began to biodegrade PCL.

Significantly, PCL is employed in the biomedical industry for “in vivo” applications such as cranial bone healing, bone fixation, and other orthopedic applications [31]. The glass transition temperature is about 60 °C. By using a catalyst like stannous octanoate, caprolactone is ring-opened polymerized to create PCL. Since PCL can be used as an implantable biomaterial but is hydrolyzed by its ester linkages in physiological settings (in the human body), which has attracted much attention. Thus, it is used for long-term implanted device development. PCL beads have been used to contain a range of pharmaceuticals for controlled release and targeted drug administration [32]. The medical field has benefited dramatically from polycaprolactone. The multifunctional uses of polycaprolactone include drug conjugates, nanoparticle coating, and pharmaceutical preparation and formulation. Besides biodegradability, biocompatibility, non-toxic, and cost-effectiveness, it can be customized by changing the molecular weight, degree of crosslinking, and crystallization. In creating scaffolds, artificial organs, and nerve regeneration, PCL and PCL-based materials have demonstrated promising outcomes [33].

To create biodegradable 3D printed scaffolds, polycaprolactone, initially synthesized in the 1930s, is currently employed. PCL possesses flexibility qualities and is employed in various materials, including films, fibers, and microparticles. PCL is widely employed for numerous drug delivery applications, such as personalized drug delivery, and has the engineering of tissue capacity. Since fused deposition modeling is the most practical technology for creating individualized scaffolds and implants, several researchers are developing biodegradable composites with the reinforcement of biodegradable fillers using PCL [34]. As discussed in PCL’s well-known regeneration capacities, PCL scaffolds can be prepared to construct artificial periodontal ligament tissue using the electrospinning technique. For regenerating natural periodontal ligament (PDL), the work carried out by researchers is to separate human periodontal ligament stem cells (PSCs) and use synthetic polycaprolactone as a vehicle for their implantation. PDL cells were created by culturing and expanding hPDLSCs, which were taken from removed human premolars. An immunofluorescent technique was used to find a PDL-specific marker called periostin. On the generated PCL, the isolated hPDLSCs were implanted. Field emission scanning electron microscopy was used to assess the scaffolds after 21 days. An MTT assay with varied cell exposure periods was used to characterize the PCL scaffold’s biological response. A cell sheet was successfully created by using PCL as a scaffold to cover dental implants and stimulate PDL cell adhesion, proliferation, and growth for the construction of biohybrid implants [35–37]. PCL has moduli between 343.9 and 364.3 MPa, tensile strengths between 10.5 and 16.1 MPa, and tensile yield strengths between 8.2 and 10.1 MPa. PCL has a 38.7 MPa compressive strength, a 297.8 to 317.1 MPa compressive modulus, and a 10.3 to 12.5 MPa compressive yield strength [38].

The practical design of medical polymer materials that come into close contact with human surroundings, such as bodily fluid, blood, and muscle, requires adequate biocompatibility, considerable absorption of degradation products, and simplicity of processing. One simple and proper technique is to use physical vapor deposition (PVD) or chemical vapor deposition (CVD) to coat antibacterial films made of polymers and antibiotics on the surfaces of artificial implants [39]. The low-energy electron beam dispersion (EBD) method can successfully be used for physical vapor deposition. With the use of polycaprolactone and the EBD method, the effective broad-spectrum antibiotic ciprofloxacin hydrochloride (CIP) was successfully transported into the composite membrane, resulting in a mechanical mixing of the original medication that did not form any new chemical bonds. Antibiotics and biopolymer materials can be physically combined to prevent the drug molecule from thermally deteriorating during high-temperature evaporation, and the resulting composite film can prevent bacterial adherence [40].

PCL is an excellent polymer for bio-implants because of its adaptable physio-chemical state, biological characteristics, and mechanical strength, which allow it to endure physical, chemical, and mechanical assaults without losing its qualities. It is also hydrophobic and degrades slowly. Because of its friendly nature and tailorable features, it has been employed in most innovative drug delivery systems and tissue engineering applications that have been used/investigated so far [41]. Polycaprolactone can be used to restore cartilage and bone. PCL is more stable under ambient settings, cheaper, and readily accessible, with a melting point of 60 degrees Celsius. It has regained prominence after the debut of FDM because of its better rheological and viscoelastic qualities over many of its aliphatic polyester rivals. Its popularity has been exceeded by faster restorable polymers with fewer perceived limitations related to long-term breakdown and intracellular resorption routes [42].

FDM-printed PCL possesses Young’s Modulus of 216 MPa, elongation at breaks of 746%, and tensile strength of 37 MPa. This allows for the development of longer-lasting degradable implants with tailorable breakdown kinetics to fit a specific anatomical region. Furthermore, PCL is stable in the body for more than 6 months and completely dissolves in 3 years, allowing it to stimulate tissue regeneration or healing and then be biodegraded without producing potentially toxic by-products [43]. PCL-based scaffolds can be employed in skin regeneration, skeletal muscle tissue regeneration, tendon regeneration, and cartilage and bone tissue restoration and regeneration. However, because pure PCL lacks the osteogenic capacity to promote bone regeneration, it is mixed with other inorganic chemicals, polymers, metal elements, and so on [44–46].

Advantages:

- Slow degradation rate: PCL has a slow rate of deterioration, which might be helpful in situations where long-term stability is required. During tissue regeneration, it can offer structural support and gradually deteriorate as new tissue develops. Controlled Release and Gradual Degradation: Polycaprolactone biopolymers can be engineered to degrade gradually, enabling controlled release of drugs or therapeutic agents over an extended period. This feature is particularly useful for drug delivery systems and tissue regeneration applications.
- Scaffold for tissue engineering: polycaprolactone biopolymer can be 3D printed into intricate scaffold structures that resemble the natural extracellular matrix. These scaffolds provide a supportive environment for cell growth and tissue regeneration, making them valuable in tissue engineering.
- Biocompatible surface modification: polycaprolactone biopolymers can be easily modified to enhance their biocompatibility. Surface modifications, such as the addition of bioactive molecules or coatings, can promote cell adhesion, proliferation, and differentiation, facilitating better integration with surrounding tissues.
- Compatibility with other materials: polycaprolactone biopolymer can be blended or combined with other biomaterials or additives to enhance its properties. For instance, the addition of ceramic particles can improve mechanical strength or bioactivity of the printed constructs.

The disadvantage of PCL includes:

- Slow degradation rate: PCL degrades very slowly, which can be a disadvantage in applications where rapid degradation is desirable. This can lead to prolonged exposure of the body to the polymer, which can cause adverse effects.
- Limited mechanical strength: PCL has relatively low mechanical strength, which can limit its use in load-bearing applications such as bone implants. This can also limit its use in tissue engineering applications where mechanical properties are important.
- Poor cell adhesion: PCL does not support cell adhesion and proliferation as well as other biodegradable polymers such as polylactic acid (PLA) and polyglycolic acid (PGA). This can be a disadvantage in tissue engineering applications where cell growth and attachment are critical.
- Hydrophobicity: PCL is highly hydrophobic, which can limit its ability to interact with biological fluids and tissues. This can affect its ability to deliver drugs or interact with cells.
- Toxicity: while PCL is generally considered biocompatible, there are concerns about the potential toxicity of PCL degradation products.

### Polylactide

One of the most popular biodegradable thermoplastic polymers, polylactide (PLA), finds use in various industries, including the biomedical and renewable sectors. Due to its sustainable, eco-friendly, and entirely biodegradable qualities, PLA has become a promising material. because of its ester linkages connecting the monomer units, this biopolymer, which is categorized as an aliphatic polyester, this biomaterial has crucial uses in the biomedical sector for a variety of uses, including suture threads, bone fixation screws, and medication delivery devices. However, PLA’s utility as a carrier and scaffold for drug delivery is currently limited [47]. Another application is in tissue engineering, where the solid-state drawing (SSD) was first successfully used to create oriented shish-kebab crystals of stereo complex poly(lactic acid) (SC-PLA) to simultaneously improve mechanical performance and biocompatibility. Environment-friendly and lightweight thermoplastic polymers based on polylactide (PLA) are gaining popularity because they decompose into components that can be converted into metabolic pathways while causing no harm to the environment [48].

Comparing the surface morphology of SC-PLA samples to that of a typical biomedical polymer with a smooth surface structure reveals a substantial difference. This is caused by the epitaxial lamellas’ uniform spacing and highly orientated fibrous backbone structure. This specific feature, which is desired for cell adhesion-growth to boost proliferation, differentiation, and activity on the surface of SC-PLA, exactly represents the microstructure of the human vascular endothelium [48]. Nanoparticles have been developed based on the hydroxyethyl starch-polylactide (HES-PLA) polymer, onto which they placed the TGF- inhibitor LY2157299 and the chemotherapy drug doxorubicin. This multimodal delivery approach seeks to stop both tumor growth and metastasis [49].

**Fig. 4:**
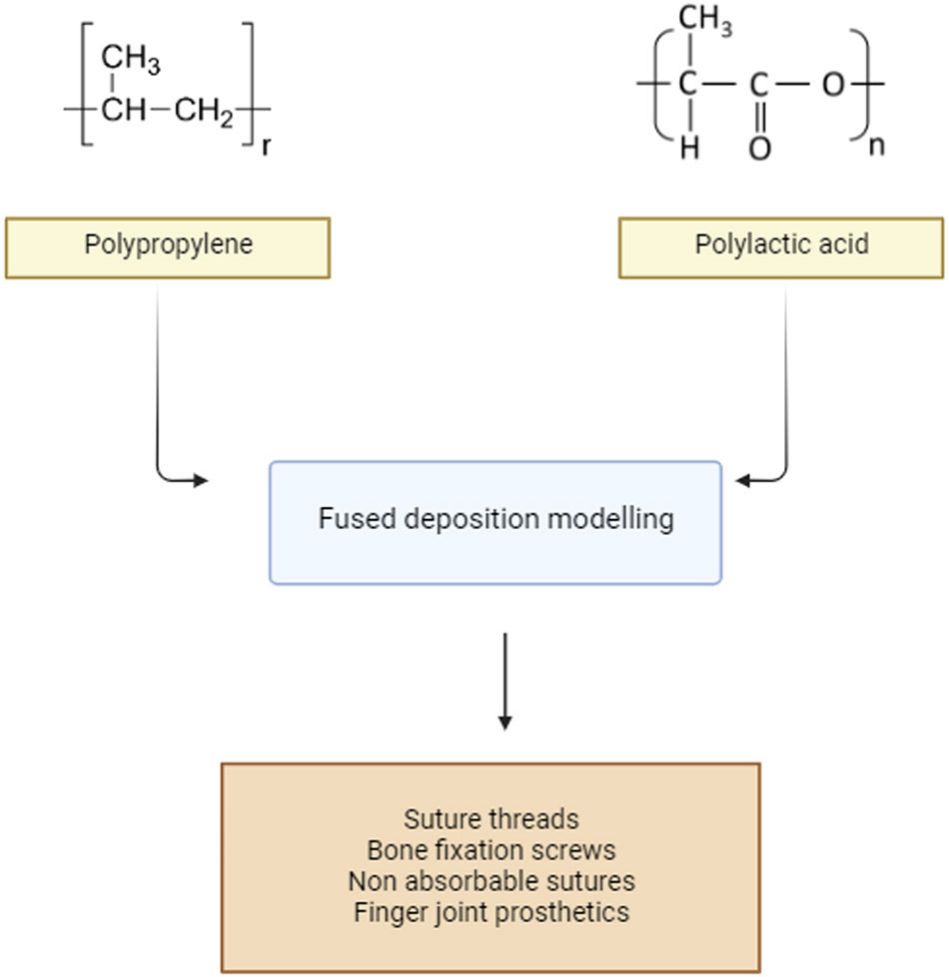
Preparation of polypropylene and poly lactide based biomedical implants

Figure 4 represents the fabrication of PP and PLA based biomedical implants. PLA is a promising material with high mechanical properties (flexural strength up to 140 MPa, Young’s modulus 5–10 GPa), remarkable optical characteristics, superior processing capabilities (with minimal shrinkage that does not cause product deformation), and 100% biodegradability. PLA is not without flaws. It is a brittle material with a Charpy impact fracture of 2.5 kJ/m^2^, a total elongation in tensile of around 3%, and progressive crystallization and enzymatic hydrolysis, which restricts its application [50].

Apart from the parts of tissues, it also aids in treating segmental bone defects. Resorbable polylactide membrane has shown promise in treating bone abnormalities in animal tests and craniomaxillofacial surgery and the results obtained were positive where one graft containment in segmental bone defects was accomplished safely and effectively using resorbable polylactide membrane, BMP-2, and autograft [51]. With a few restrictions including a slow breakdown rate, hydrophobicity, and low impact toughness related to its use, PLA is an eco-friendly non-toxic polymer with properties that permit use in the human body. To improve PLA’s thermal characteristics and avoid deterioration, crosslinking, copolymerization, and recrystallization are utilized. PLA fiber shows good self-extinguishing characteristics, although it is not a non-flammable polymer [52, 53].

**Fig. 5:**
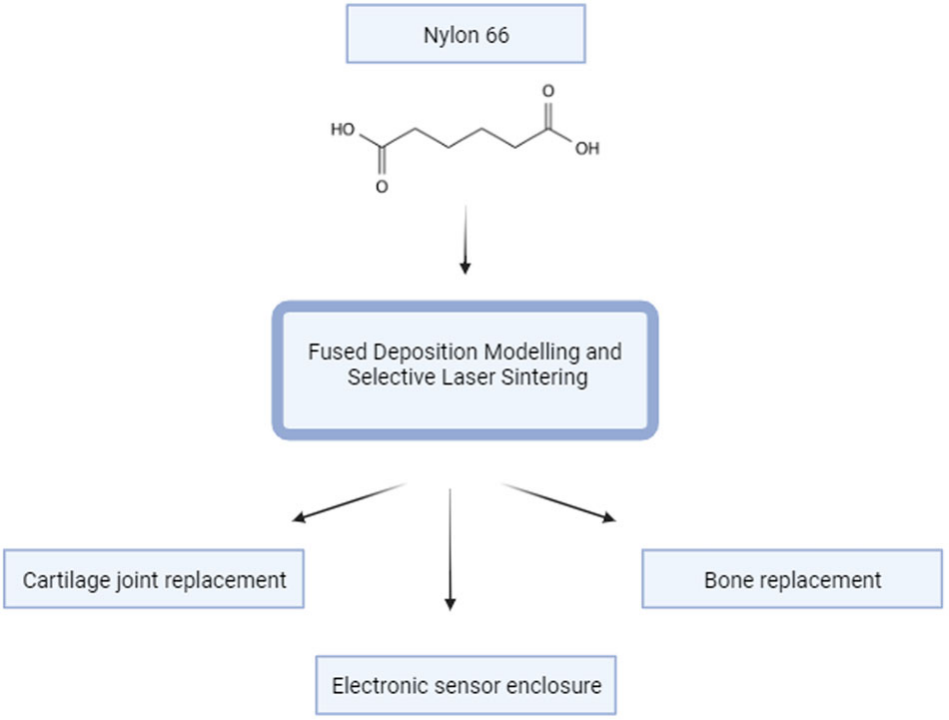
Process of preparation of Nylon 66 based applications by 3D printing

**Fig. 6:**
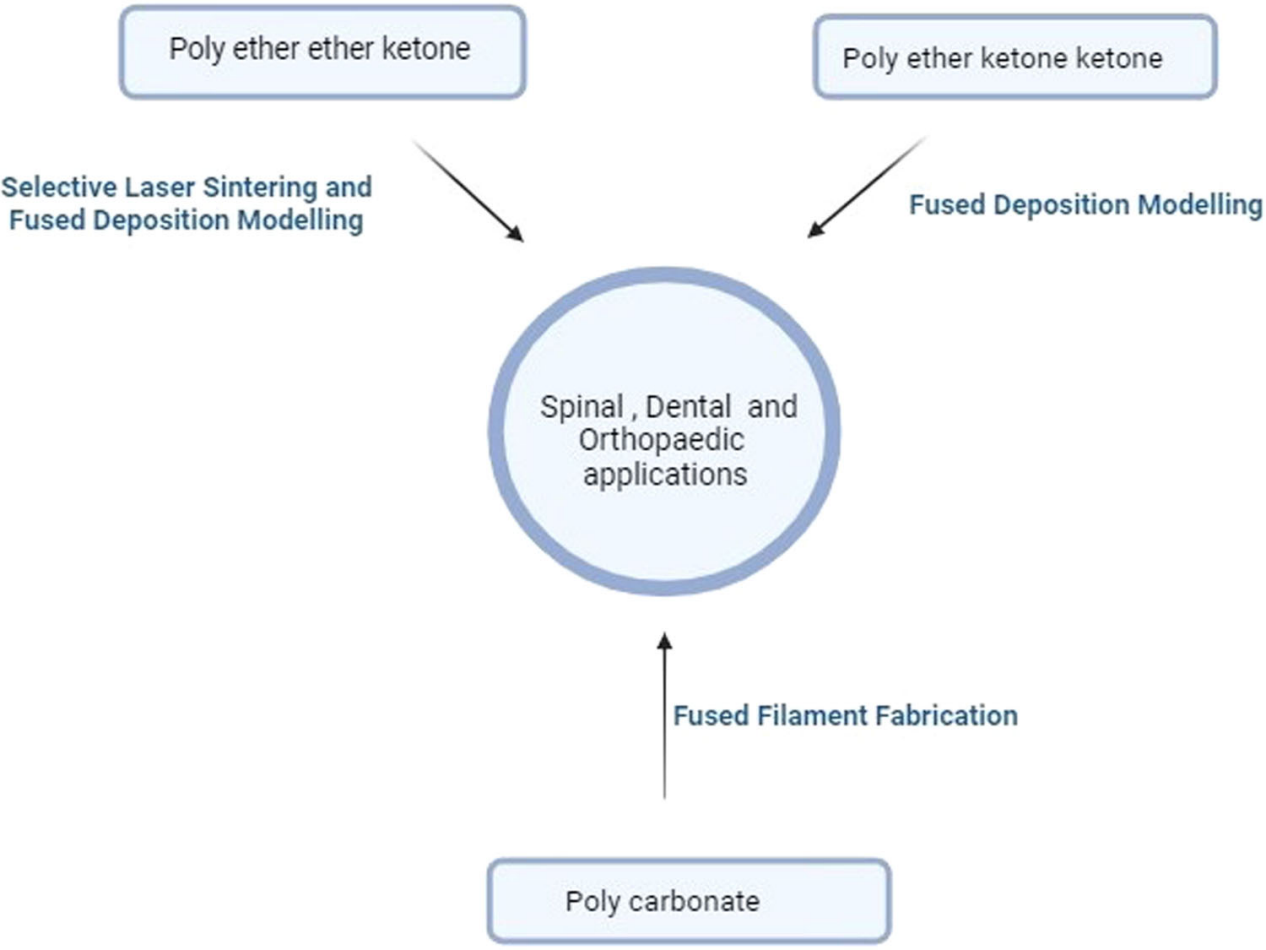
PEKK, PEEK, and PC-derived biomedical applications and their printing technology

**Fig. 7:**
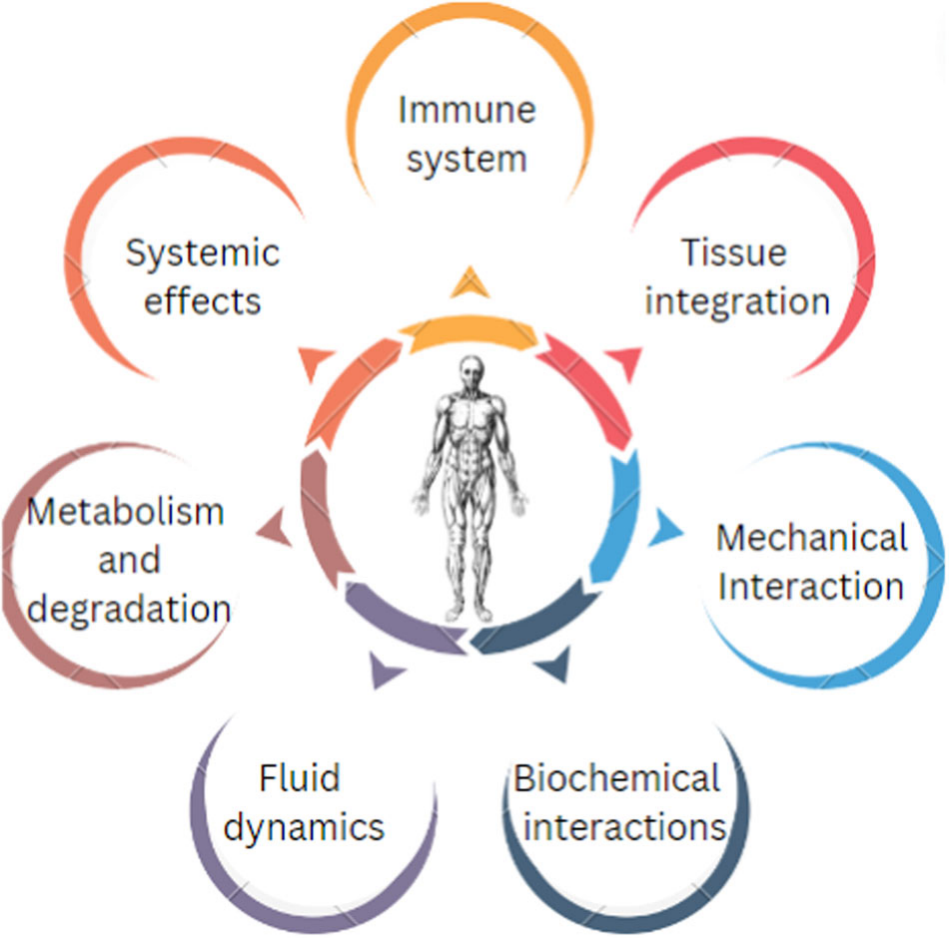
Aspects and interactions that needs to be looked into while designing and selecting a material for implant

The Tg of PLA affects its physical characteristics, including density, heat capacity, mechanical, and rheological characteristics. Because of the variations in polymer chain mobility at and above Tg, Tg is one of the most crucial parameters for amorphous PLA (Figs. 5–7). Tg and Tm are crucial physical factors in forecasting PLA behavior in semi-crystalline PLA. The melting enthalpy of an enantiopure PLA with 100% crystallinity (H°m) is 93 J/g. The densities of crystalline PLA are 1.290 g/ml and amorphous PLA 1.248 g/ml, respectively. The reported densities of L-lactide are 1.36 g/cm^3^, meso-lactide with 1.33 g/cm^3^, PLA is crystalline and is 1.36 g/cm^3^, and PLA is amorphous and 1.25 g/cm^3^ [54]. PLA must have an ultra-high molecular weight to be processed into solid fibers. PLA is a thermally less stable polymer with poor physical properties and a high production cost [55]. By developing composites or nanocomposites using PLA as a matrix and additives like fillers, nucleates, nano compounds, or fibers as additives, it is possible to change PLA’s mechanical, thermal, and processing properties. The tensile strength increases when additives are present, such as with the addition of iron powder. In contrast, the magnesium powder somewhat increases the ductility of the composite material (from 2.0 to 2.5%) at the expense of a slight drop in strength [56].

PLA is widely utilized in 3D printing. 3D printed PLA has been utilized to make BioSource-based implants and devices, and its copolymers have also been employed in the manufacture of screws and scaffolds, in addition to serving as a drug carrier medium. It has a tensile strength of around 50 to 89 MPa, a flexural strength of ~120–150 MPa, a flexural modulus of ~3.5–5 GPa, and a density of ~1.2 grams per ML with an elongation per percentage of 2 PLA is 100% biodegradable, may be recycled up to 8 times, and is compostable up to 10. Young’s modulus is 3–5 GPa. However, it is not very tough and has a slow degradation rate and is also hydrophobic. Their clinical applications are sometimes hampered by their high hydrophobicity and poor water uptake, resulting in a slow hydrolytic degradation rate.

FDM 3D printed PLA composites provide mechanical qualities and functions that would be difficult to accomplish with PLA alone. PLA is frequently reinforced with fibers like hemp, jute, and bamboo [57]. PLGA poly (lactic-co-glycolic acid) is widely used in bone tissue engineering. It is highly biocompatible, biodegradable, and has no bio toxicity. Extrusion printing is used to create most of the 3D scaffolds that have excellent biological properties and hence play an essential role in tissue engineering. Fused deposition modeling technology is also used. FDM results in the development of devices with good stability and slow-release characteristics [58]. PLA filaments are used as containers, for low-cost rapid prototyping, fixtures, and jigs. PLA can also be combined with other flexible polymers that function as plasticizers to increase mechanical strength while decreasing brittleness. It is a more sustainable alternative to petrochemical-derived goods since it is generated from lactides, which are produced by the fermentation of agricultural byproducts such as corn starch or other carbohydrate-rich substances such as maize, sugar, or wheat [59].

Advantages of PLA:

- Applications in biomedicine: PLA has undergone substantial research for uses in biomedicine, including drug delivery systems, scaffolds for tissue engineering, and biodegradable implants. It is appropriate for these applications due to its biocompatibility and capacity to break down into non-toxic components.
- Tailored mechanical properties: PLA can be customized to have a wide range of mechanical properties, allowing for flexibility in meeting specific application requirements. It can be made rigid or flexible to suit different tissues or structures.
- Optical clarity: PLA exhibits good transparency and clarity, which is beneficial for applications requiring visual monitoring, such as tissue scaffolds or drug delivery systems.
- Reduced inflammation risk: PLA has a lower risk of triggering inflammation compared to some other materials, making it suitable for implantable devices or biodegradable sutures.
- Easy sterilization: PLA is compatible with various sterilization methods like autoclaving, gamma irradiation, or ethylene oxide treatment, ensuring effective sterilization of 3D printed PLA biomedical devices.

Disadvantages of PLA can be summarized as:

- Poor mechanical properties: PLA has poor mechanical properties compared to other biodegradable polymers, such as polyglycolide (PGA) and polycaprolactone (PCL). This limits its use in load-bearing applications such as bone implants.
- Limited solubility: PLA has limited solubility in common solvents, which can make it difficult to process and fabricate into various forms, such as nanoparticles or scaffolds.
- Brittleness: PLA can become brittle over time due to hydrolysis, which can limit its durability in some applications.
- Acidic degradation products: PLA degrades into acidic products, which cause inflammation and tissue can damage in vivo. This can be a concern in some applications, such as tissue engineering and drug delivery.

### Nylon

Nylon is a synthetic polyamide polymer with great tensile strength, excellent flexibility, and low tissue reactivity [60]. This polymer satisfies biomedical needs by producing sutures, catheters, and dentures due to its biocompatibility, chemical stability, tunable mechanical qualities, and 3D printability. Nylon is functionalized and hybridized with other materials to make skin dressings. Nylon composites have been the subject of intensive tissue engineering research in recent years as a potential replacement for metallic implants with a suitable bioactivity potential for bone formation.

Permanent multifilament fiber structures constructed of nylon 6 or nylon 6,6 are used to create nylon skin sutures, which must have a high level of strength, a thin thickness, and flexibility. Natural polymers may be added to the surface of nylon sutures to boost their antibacterial ability because they are used to close wounds to stop the spread of infection and speed up wound healing. Chitosan and hyaluronic acid are two examples of natural polymers that are great additions since they have an antibacterial impact [61]. Nylon is the primary important synthetic polyamide with clinical applications. Suture materials make use of their high tensile strength. Polyamide block copolymers with soft segments for improved elasticity combined with the flexibility of polyurethanes and the strength of nylon make it the preferred material for angioplasty balloon catheters [62].

Catheters made of nylon are also effective. For cardiac catheters, nylon’s capacity to create tiny tubes with acceptable dimensional tolerance is essential. Because of the limited vein size and nylon’s elasticity, catheters can withstand severe cyclic compressive pressure while the heart is beating and still perform its intended function [63]. Catheter balloons can also be made from nylon polymer, which can be devised for dilating and clearing blockages in blood vessels, enhancing drug delivery, and the delivery and expansion of stents for cardiovascular disorders. Nylon based osteo applications are also possible however the application is restricted with mess weight loaded operations. Nylon in combination with rayon and cotton has also been formulated to be used as nasopharyngeal swabs as an intervention to overcome shortage during SARS Covid testing [64, 65].

Advantages of Nylon 66:

- Bioresorbable: nylon biopolymer can be formulated to be bioresorbable, meaning it can degrade naturally over time within the body. This property is particularly advantageous for temporary implants or scaffolds, as they can gradually degrade as new tissue forms.
- High tensile strength: nylon biopolymer exhibits high tensile strength, allowing it to withstand significant mechanical forces. This makes it suitable for applications that require robust and durable materials, such as load-bearing implants or surgical tools.
- Excellent biocompatibility: nylon biopolymer has shown excellent biocompatibility, minimizing the risk of adverse reactions or tissue rejection. This makes it suitable for use in direct contact with biological systems, such as implants or tissue engineering scaffolds.
- Chemical resistance: nylon biopolymer is resistant to a wide range of chemicals, providing protection against degradation or damage in harsh biological environments. This property is crucial for long-term stability and functionality of biomedical devices.
- Versatility in processing: nylon biopolymer can be processed using various 3D printing techniques, including selective laser sintering (SLS) or fused deposition modeling (FDM). This versatility in processing methods allows for the creation of intricate and complex structures with high precision.

The disadvantage of using Nylon includes:

- Non-biodegradability: nylon is non-biodegradable, which can limit its use in applications where the polymer needs to be metabolized or eliminated by the body. This can lead to long-term accumulation of the polymer in the body, which can cause adverse effects.
- Limited solubility: nylon has limited solubility in common solvents, which can make it difficult to process and fabricate into various forms, such as nanoparticles or scaffolds.
- Limited mechanical properties: nylon has limited mechanical properties compared to other polymers, such as polycaprolactone (PCL) and polyglycolide (PGA). This limits its use in load-bearing applications, such as bone implants.
- Moisture sensitivity: nylon is highly moisture-sensitive, which can lead to swelling and degradation over time. This can limit its durability in some applications.

### Polyether ether ketone and polyether ketone ketone

A member of the polyaryletherketone (PAEK) family, polyether ether ketone (PEEK) is an organic thermoplastic colorless polymer [66]. PEEK is rarely printed in 3D due to the requirement of higher melting temperatures, the scarcity of suitable feedstock, worries about poor adhesion between layers, and labor-intensive and expensive processing steps.

However, the material has demonstrated that it is acceptable for use in the following applications: dental, oral, and craniofacial/maxillofacial [67]. This polymer can also be used for orthopedic implants such as hip joints [66], replacements for broken bones [68, 69], and scaffolding [70] because it satisfies the requirements for more excellent mechanical properties, chemical stability, biological stability, and biocompatibility. Like PEEK, this polymer’s applications have expanded to include spine surgery and Oro-maxillofacial procedures [71]. Polyetherketoneketone, another polymer in the PAEK family, is used for similar purposes. PEKK has strong osteointegration and excellent biocompatibility. Like PEEK, this polymer’s applications have expanded to include spine surgery and Oro-maxillofacial procedures [72].

High-performance polymers are solid, have outstanding mechanical and thermal resistance, and are much lighter. They are desirable for biomedical applications due to these qualities. PEEK and PEKK 3D printers must have a heating plate that can reach at least 230 °C, an injection molding that can reach 350 °C, and a closed chamber due to the materials’ high melting point (350 °C). However, this quality grants 3D-printed high-performance polymers excellent heat resistance, enabling them to go through steam sterilization without softening [73]. PEEK has a great application in bone tissue engineering and craniofacial engineering because of its properties, such as biocompatibility, radiolucency, and low moisture absorption. With Young’s modulus of 3.6 GPa and tensile strength of 100 MPa, PEEK possesses good mechanical properties. Moreover, because of these properties, they are widely used for various medical purposes. Although PEEK has excellent properties and an elastic modulus like natural bone, it has poor assimilation with nearby bone tissue after implantation [74].

In terms of mechanical durability, chemical resistance, and thermal stability, PEKK biomaterials perform better than many thermoplastic composites. They possess higher mechanical and physical qualities, such as compressive strength and melting temperature, than other polymeric materials. PEKK is an effective restorative material due to its fracture resistance and suitable stress absorbance. They share PEEK’s bone-like elastic modulus. They feature good wear resistance, a low density, a high-strength-to-weight ratio, and a low elastic modulus. They have numerous biomedical uses because of their great mechanical strength and the presence of the second ketone group, which enables additional surface modification of its surface [75]. As a biomaterial with qualities appropriate for dental and medical applications, PEKK is being used more frequently. The PEKK has numerous uses in implant, restorative, and prosthetic dentistry. The PEKK is a material that shows promise for use in cranial and orthopedic implants [76].

PEEK is very flexible and can sustain high degrees of plastic deformation in traction and compression despite its macromolecular stiffness. Its stress-strain behavior exhibits a clear flow transition. In compression, PEEK has yield stress that is 30 to 40% greater than in tension. PEEK is an aromatic thermoplastic with a high glass transition temperature (Tg) of 143 °C due to the extraordinary stiffness of its macromolecular structure. Despite its great macromolecular stiffness, polymer chains may be arranged in crystalline and amorphous domains due to their planar zig-zag molecular shape. PEEK’s long-term mechanical characteristics are heavily influenced by its glass transition temperatures and melting point. The stiffness, strength, and toughness of PEEK at operating temperatures have boosted its use in surgical biomedical implants [77].

Despite its advantageous biomaterial characteristics, PEEK is rather bioinert. PEEK’s mechanical characteristics are governed by its crystallinity level, and it has previously been demonstrated that increasing crystallinity can boost elastic properties and yield strength while decreasing toughness. Crystallinity is influenced by both thermal history and material cooling time. As a result, the level of the crystalline phase of PEEK in the injection molding process is normally regulated by mold temperature [78].

Advantages:

- Biocompatibility: PEEK is highly compatible with the human body, making it suitable for medical implants and devices. It integrates well with surrounding tissues and has bone-like properties.
- Mechanical properties: PEEK has excellent mechanical properties, including strength, stiffness, and fatigue resistance. This makes it ideal for load-bearing applications like orthopedic implants.
- Wear and abrasion resistance: PEEK is highly resistant to wear and abrasion, ensuring long-term durability in biomedical applications. It can withstand the mechanical stresses and friction within the body.
- Customizability: 3D printing allows for the creation of personalized implants and devices. With PEEK, custom-made implants can be designed to fit an individual’s unique anatomy, leading to better surgical outcomes.
- Processing and manufacturing: PEEK is easily processed and manufactured using 3D printing techniques. Compared to traditional methods, 3D printing offers more design freedom and flexibility.
- Potential for improved osseointegration: ongoing research aims to enhance the integration of PEEK with bone tissue. Surface modifications and the inclusion of bioactive substances are being explored to improve osseointegration.

Disadvantages of PEEK includes:

- Non-biodegradability: PEEK is non-biodegradable, which can limit its use in applications where the polymer needs to be metabolized or eliminated by the body. This can lead to long-term accumulation of the polymer in the body, which can cause adverse effects.
- Cost: PEEK is relatively expensive compared to other polymers used in biomedical applications, which can limit its use in some settings.
- Limited solubility: PEEK has limited solubility in common solvents, which can make it difficult to process and fabricate into various forms, such as nanoparticles or scaffolds.
- Brittleness: PEEK can become brittle over time, especially under certain conditions such as high temperatures or exposure to radiation. This can limit its durability in some applications.
- Imaging interference: PEEK can interfere with medical imaging techniques such as X-rays, which can limit its use in applications where imaging is needed to monitor the progress of treatment.

### Glycolic acid

The earliest synthetic absorbable sutures were made of polymers; polyglycolic acid (PGA) and poly lactic-co-glycolic acid (PLGA) have been used to create biodegradable staples, screws, meshes, and stents in addition to being used as bioabsorbable suture materials. The lactic acid to glycolic acid ratio, molecular mass, and storage temperature of the PLGA polymer used for synthesis are the primary determinants of the physicochemical characteristics of PLGA particles. However, most of the particle properties, such as size distribution and particle size, are hugely affected by the synthesis such as the addition of encapsulated substance [79].

Studies in PGA are increasing due to its biodegradability and environmentally acceptable qualities. In contrast to other applications, PGA is primarily used in biomedical applications because PGA degradation produces glycolic acid, a natural metabolite. However, due to PGA’s rapid degradation and insolubility in most solvents, there needs to be more research on PGA polymers in biomedical applications [79]. Since PLGA is biocompatible and produces safe, non-toxic degradation products, they are suitable for many pharmaceutical and medical applications. The hydrolytic breakdown of lactic and glycolic acids, which are ultimately eliminated from the body as CO2 and water, occurs during the degradation process of PLGA-based homo and copolymers [80].

Researchers have focused much emphasis on polylactic acid-glycolic acid (PLGA), a typical biodegradable polymer. Devices made of PLGA are often employed in the biomedical industry. The mechanical, biocompatibility and tunable degrading qualities of PLGA make it a viable choice for various machining applications. Diverse techniques have been used to create PLGA-based polymers that have been used to mend bone tissue in various body areas. PLGA is often dissolved in organic solvents like acetone and 1,4-dioxane to make printing ink comply with the 3D printing processing mode. Its biodegradability as an artificial bone transplant is directly tied to bone growth, which is very important. The LA: GA ratio, molecular weight, and end-group of the molecular chain all impact the rate at which PLGA scaffolds degrade [79].

PLGA poly (lactic-co-glycolic acid) is widely used in bone tissue engineering as stated in the previous section. It is highly biocompatible, biodegradable, and has no bio toxicity. Extrusion printing, low-temperature 3D printing, is used who create most of the 3D scaffolds that have excellent biological properties and hence play an essential role in tissue engineering. Fused deposition modeling technology is also used. FDM leads to devices with good stability and slow-release characteristics [81]. PLA filaments are used as containers, and pastry molds, for low-cost rapid prototyping and in the automotive industry as print tools, fixtures, and jigs. Hydrophilicity, poor mechanical properties, and shorter half-life limit its application in load-bearing and drug-delivery applications [79].

Advantages:

- Customizability: 3D printing enables the creation of personalized implants and devices. By using glycolic acid or similar materials, it may be possible to fabricate custom-made implants tailored to an individual’s specific needs and anatomy.
- Potential for controlled release: glycolic acid and related materials have been studied for their ability to enable controlled release of drugs or therapeutic agents. Incorporating this feature into 3D printed biomedical devices could allow for targeted and sustained delivery of medications or other substances.
- Ease of processing: glycolic acid and similar materials can be processed using 3D printing techniques. This ease of processing allows for the creation of complex structures and intricate designs, which may be advantageous in developing biomedical devices with specific functionalities.

Disadvantages of glycolic acid includes:

- Hydrophilicity: GA is highly hydrophilic, which can limit its ability to interact with hydrophobic drugs or tissues. This can affect its ability to deliver drugs or interact with cells.
- Poor mechanical properties: GA has poor mechanical properties compared to other biodegradable polymers, such as poly(lactic-co-glycolic acid) (PLGA) and polycaprolactone (PCL). This limits its use in load-bearing applications such as bone implants.
- Acidic degradation products: GA degrades into acidic products, which can cause inflammation and tissue damage in vivo. This can be a concern in some applications, such as tissue engineering and drug delivery.
- Limited solubility: GA has limited solubility in common solvents, which can make it difficult to process and fabricate into various forms, such as nanoparticles or scaffolds.
- Sterilization: GA is sensitive to high temperatures, which can limit its ability to be sterilized using common methods, such as autoclaving.
- Short half-life: GA has a short half-life, which can limit its effectiveness as a drug delivery system.

### Acrylonitrile butadiene styrene (ABS)

Acrylonitrile butadiene styrene is one of the most widely used and versatile materials used for 3D printing. It is a high-impact engineering thermoplastic and amorphous polymer. Its three monomers are acrylonitrile, butadiene, and styrene and its numerous advantageous physical features are high insulating capabilities, weldability, and resilience to abrasion and strain. ABS is also mechanically tough, long-lasting, and consistent throughout time. It is resilient to frequent changes in temperature and is highly durable. The polymer, in addition to being chemically resistant, has a high surface brightness and superb surface quality. It is suitable for usage in the automotive, electronics, building and construction, home appliances, and transportation industries due to its strong impact strength and heat performance [82]. An engineering thermoplastic that is amorphous and resistant to impacts is acrylonitrile butadiene styrene. It is used to ensure minimal warping and consistent interlayer adhesion. It possesses many advantageous characteristics, such as solid stiffness, superior insulating qualities, impact resistance at low temperatures, and strain. Aside from these characteristics, ABS is also long-lasting, resilient, and mechanically robust. The polymer possesses a good surface quality, a high surface brightness level, and is chemically resistant. Due to its excellent purity, uniformity, and low residual monomers, ABS has a variety of uses in the medical sector [83, 84].

Acrylonitrile styrene acrylate (ASA) is an amorphous synthetic thermoplastic that works best in material extrusion printing. They are highly impact-resistant and have excellent UV stability. The mechanical properties of ASA are excellent, with tensile strengths ranging from 35.0 to 50.5 MPa and densities ranging from 1.06 to 1.09 g/cc. They can also be thought of as a heat-resistant alternative to ABS. However, due to its low biocompatibility and toxicity, it is not widely used in the medical or pharmaceutical fields. ABS was one of the first plastics for 3D printing that was frequently employed in biological applications. It is a more durable and highly flexible copolymer based on polybutadiene. ABS can resist temperatures as low as 20 °C and as high as 80 °C and have a melting point of 105 °C, making it a desirable material for use in FDM and SLA systems [84]. On the other hand, ABS is highly sensitive to UV and contracts when exposed to air, it is frequently utilized in engineering cartilage and the nucleus pulposus. Adding to this, Acrylonitrile butadiene styrene is biocompatible for up to 30 days after being in contact with the human body. Since it is adaptable, it is used in medical equipment such as medical masks and valves for ventilators [85].

Advantages of ABS:

- Chemical resistance: ABS demonstrates good resistance to chemicals, making it suitable for applications involving contact with various substances, such as chemical processing or laboratory environments.
- Electrical insulation: ABS has excellent electrical insulation properties, making it advantageous in applications requiring electrical components or insulation, such as bioelectrodes or electronic devices.
- Post-processing options: ABS is easily post-processed, allowing for modifications or enhancements after 3D printing. It can be sanded, painted, or smoothed to achieve desired surface finishes or esthetics.
- Low shrinkage: ABS exhibits relatively low shrinkage during 3D printing, reducing the likelihood of warping or distortion in complex shapes or large-scale prints. This improves dimensional accuracy and overall print quality.
- Recyclability: ABS is a recyclable material, contributing to sustainability goals and minimizing waste in the production and use of 3D printed biomedical devices.

The disadvantage of ABS includes:

- Non-biodegradability: ABS is non-biodegradable, which can limit its use in applications where the polymer needs to be metabolized or eliminated by the body. This can lead to long-term accumulation of the polymer in the body, which can cause adverse effects.
- Poor biocompatibility: ABS is generally not biocompatible and can cause inflammation and tissue damage in vivo. This limits its use in applications where the polymer needs to be in contact with biological tissues.
- Limited solubility: ABS has limited solubility in common solvents, which can make it difficult to process and fabricate into various forms, such as nanoparticles or scaffolds.
- Limited mechanical properties: ABS has limited mechanical properties compared to other polymers, such as polycaprolactone (PCL) and polyglycolide (PGA). This limits its use in load-bearing applications, such as bone implants.
- Sensitivity to UV light: ABS is sensitive to UV light, which can cause degradation and discoloration over time. This can limit its durability in some applications.

### Polycarbonates (PC)

A class of thermoplastic polymers known as polycarbonates has carbonate groups in their chemical compositions. Although polycarbonate and polymethyl methacrylate (PMMA) have properties in common, polycarbonate is more robust, expensive, and useful across a wider temperature range. Since it exhibits exceptional compatibility with specific polymers, PC/ABS, PC/PET, and PC/PMMA, PC/PET [86] is frequently used in blends. Exceptional mechanical strength, superior toughness, and good optical transparency are examples of engineering qualities. Due to these features, PC is widely used in electronics, building, data storage, and the automotive sector, apart from biomedical applications [87]. A recent effort aimed at synthesizing low-cost biodegradable biopolymers from recycled polycarbonate waste by polycondensation technique achieved polymers having good biocompatibility in both in vitro and in vivo systems and supporting circular economy [88, 89].

Polycarbonates are a unique and efficient class of high-heat polymers with high toughness. BPA polycarbonates are well-known for their optical clarity, high-impact resistance, and ductility at or below room temperature. Their glass transition temperatures (Tg) range between 140 and 155 °C. This material has a high modulus, dielectric strength, and tensile strength when compared to other amorphous thermoplastics. Most of the amorphous polymers become stiff and brittle below their glass transition temperature, whereas polycarbonates retain their ductility [90].

It is observed that the addition of carboxylic acid-functionalized polycarbonate block copolymers to commercially available bone cement improved antibacterial activity. In comparison to a commercially available gentamicin-containing cement control that demonstrated 70 days of activity, the top-performing polymers demonstrated 259 days of sustained antimicrobial release against S. aureus and 147 days against P. aeruginosa, while in vitro gentamicin release was increased by eight times [91]. In addition, the total porosity was enhanced by three times, from 4.3 to 12.5%, while bone cement’s mechanical strength, functionality, and osteoblastic biocompatibility were all preserved. polycarbonates functionalized with carboxylic acids are a promising class of bone cement additives [92].

The mechanical properties of polycarbonate can vary widely. For instance, although poly (ethylene carbonate) exhibits properties resembling rubber, poly (cyclohexene carbonate) is brittle at ambient temperature. Applications of polymers require an understanding of how they respond mechanically to external forces. In polycarbonates, the tensile modulus and Tg are closely connected. Polymers’ elongation at break and impact strength are two additional essential mechanical properties [93, 94]. Poly (propylene carbonate) is superior to polycarbonate in impact strength and elongation at break (cyclohexene carbonate). Although poly (cyclohexene carbonate) has higher mechanical properties than polycarbonates with low Tg in terms of tensile modulus and strength, its usage is limited by its brittleness (lower impact strength and elongation at break) [95]. Unlike polycarbonates made of bisphenol, bacteria can break down other polycarbonates. Polycarbonate’s structure, morphology, molecular weight, and surface characteristics affect how easily it degrades. Poly (propylene carbonate) breaks down by random chain-breaking mechanisms in air, water, and soil without releasing harmful chemicals. The poly (propylene carbonate) biodegradation rate has also been reported to be substantially influenced by environmental factors [96].

Hydrophophilicity is a crucial feature of polycarbonates that makes them a polymer with potential biological uses, including polymeric scaffolds for tissue regeneration and drug delivery systems [97, 98]. Adding functional groups to the polymer chain of polycarbonate, such as alkyl, –OH, –NH2, –COOH, and –COOR, can change the material’s hydrophilicity or hydrophobicity [99]. A potent technique for enhancing bioavailability and reducing the negative effects of anticancer drugs and chemotherapy is the use of stimulus-responsive nanostructures. A unique dual pH-responsive micellar nano platform (DOX + LAP-M) based on polycarbonate-doxorubicin conjugate micelles was employed to generate the anticancer drug lapatinib [100, 101].

For several of these applications, biocompatible polycarbonate is a promising candidate. Due to ISO and USP Class VI certifications, it boasts several exceptional qualities for use in the health industry, especially those in direct contact with humans. FFF, one of the most adaptable AM technologies, can print this filament-like polymeric substance. Nevertheless, despite PC’s high degree of biocompatibility, its employment in structural applications may call for doping with other substances that enhance mechanical performance [96]. A high-strength biomaterial with a temperature tolerance of 150 degrees Celsius, PC can tolerate physical deformation. The performance and printing resistance of PC can be impacted by moisture absorption from the air. PC has recently been used as a scaffold for medical applications, due to its adjustable porosity (from 1% to 30%) and mechanical properties, including yield stress, elastic modulus, and elongation at break values of 50 MPa, 2250 MPa, and 8.3%, respectively.

However, it should be noted that outstanding mechanical properties deteriorate as the porosity of the scaffold increases [102]. And when it comes to medical devices that deliver drugs and surgical instruments, polycarbonates are in high demand. One example is the load-bearing interior components of self-medication devices, especially those used with viscous or high-volume medications. The use of single-use sterile instruments in surgery is currently popular because it lowers the possibility of infection. Additionally, a solid thermoplastic polymer has the potential to be more effective than metal.

Advantages:

- Biocompatibility: polycarbonate biopolymer has the potential to be biocompatible, meaning it is well-tolerated by the human body. This makes it suitable for medical implants and devices.
- Strength and durability: polycarbonate is known for its strength and durability, making it a desirable material for biomedical applications. 3D printed polycarbonate biopolymer can provide strong and long-lasting components for various medical devices.
- Transparency: polycarbonate has excellent optical properties, including high transparency. This can be advantageous in applications that require visual monitoring or transparency, such as medical imaging or transparent surgical guides.
- Chemical resistance: polycarbonate biopolymer can exhibit good resistance to chemicals, which is beneficial in applications that involve exposure to various substances or sterilization processes.
- Customizability: 3D printing allows for the customization and personalization of biomedical devices. Polycarbonate biopolymer can be 3D printed to create complex and tailored structures, offering flexibility in design and functionality.

The disadvantage of using Polycarbonate includes:

- Non-biodegradability: polycarbonates are non-biodegradable, which can limit their use in applications where the polymer needs to be metabolized or eliminated by the body. This can lead to long-term accumulation of the polymer in the body, which can cause adverse effects.
- Poor mechanical properties: polycarbonates have poor mechanical properties compared to other polymers, such as poly(lactic-co-glycolic acid) (PLGA) and polycaprolactone (PCL). This limits their use in load-bearing applications such as bone implants.
- Limited solubility: polycarbonates have limited solubility in common solvents, which can make them difficult to process and fabricate into various forms, such as nanoparticles or scaffolds.
- Sensitivity to UV light: polycarbonates are sensitive to UV light, which can cause degradation and discoloration over time. This can limit their durability in some applications.
- Hydrolytic degradation: polycarbonates are susceptible to hydrolytic degradation, which can cause the polymer to break down over time. This can limit their use in applications where long-term stability is important.
- Bisphenol A: some polycarbonates are made using bisphenol A (BPA), a potential endocrine disruptor linked to adverse health effects. This can limit the use of BPA-containing polycarbonates in some applications.

### Polyamide

Another critical factor that it processes in biomaterial development is surface roughness. After being implanted, a material’s capacity to adapt and promote the surrounding tissue’s growth correlates with how rough its surface is. A rougher surface topography may significantly impact a substrate’s surface energy, improving cellular response and increasing cell adhesion and proliferation. And the surface roughness of a polyamide composite with the addition of the filler mixture is improved. A recent study also shows that Polyamide composite with 40% filler is the most promising material for use as a medical implant due to its higher flexural strength and modulus.

PA has already demonstrated good biocompatibility with various human cells and tissues, most likely due to its chemical structure and active groups resembling collagen protein. Thus, applications involving biomaterials use this material frequently. Since the amide groups in PA macromolecules establish potent hydrogen bonds with one another, PA possesses excellent mechanical characteristics. As a polar polymer with high polarity, PA has a relatively high affinity for and may form hydrogen bonds with nano-sized apatite [103]. Like the advantage it also possesses certain disadvantages that reduces its use such as it is absorbing water, resulting in lower mechanical properties.

Also, PAs are susceptible to strong bases and acids. Shrinkage percentages in cast applications are also high. To overcome this challenge, many researchers focused their efforts and significantly improved the properties of Polyamide through reinforcement methods. Reinforcements are typically made by adding solid lubricants, fillers, and additive materials to improve the base metal’s properties, such as strength, stiffness, and conductivity. It improves the material’s ductility, scratch resistance, and toughness [104]. Polyamides are widely used in SLS technology. Furthermore, because of their great biocompatibility, they are used to produce parts that encounter the skin. Polyamides, in addition to possessing a tensile strength of 10 MPa, have good stability, stiffness, flexibility, and shock resistance [105]. Polyamides and hydroxyapatite have recently been combined to create porous scaffolds with great load-bearing capacity for bone regeneration [106].

Drug delivery systems frequently employ polymer polyamide. Modern polymer chemistry has opened new possibilities for drug delivery in the human body [107]. Stabilization sustained release, and localized target site action is the advantages of using polyamide polymers in the drug delivery system [107]. Utilizing water-soluble polymer conjugates in drug delivery systems has several numbers of advantages, including improved drug pharmacokinetics, enhanced drug buildup at the target site, and minimal toxicity to critical organs, tissues, or cells but the main barrier preventing the clinical application of this is their inability to maintain integrity in the blood [108]. Sensitivity to hydrolysis limits the use of polyamide in biomedical applications.

Advantages:

- Biocompatibility: polyamide biopolymers can be designed to be biocompatible, ensuring their safe interaction with biological systems.
- Mechanical strength: polyamide biopolymers can exhibit good mechanical strength, providing durability and stability to 3D printed biomedical devices.
- Versatile design options: 3D printing allows for the creation of complex and customized designs. Polyamide biopolymers can be 3D printed into intricate structures, enabling the fabrication of personalized implants or devices.
- Chemical resistance: polyamide biopolymers may possess good resistance to chemicals, making them suitable for applications involving exposure to various substances or sterilization processes.
- Lightweight nature: polyamide biopolymers are generally lightweight, which can be advantageous for applications where weight reduction is desired, such as in wearable medical devices.

The disadvantage of Polyamides includes:

- Poor biocompatibility: polyamides are generally not biocompatible and can cause inflammation and tissue damage in vivo. This limits their use in applications where the polymer needs to be in contact with biological tissues.
- Non-biodegradability: some polyamides are non-biodegradable, which can limit their use in applications where the polymer needs to be metabolized or eliminated by the body. This can lead to long-term accumulation of the polymer in the body, which can cause adverse effects.
- Limited solubility: polyamides have limited solubility in common solvents, which can make them difficult to process and fabricate into various forms, such as nanoparticles or scaffolds.
- Limited mechanical properties: polyamides have limited mechanical properties compared to other polymers, such as polycaprolactone (PCL) and polyglycolide (PGA). This limits their use in load-bearing applications, such as bone implants.
- Sensitivity to hydrolysis: polyamides are susceptible to hydrolytic degradation, which can cause the polymer to break down over time. This can limit their use in applications where long-term stability is important.
- Potential toxicity: some polyamides, such as nylon-6,6, are made using toxic chemicals, such as hexamethylene diamine and adipic acid. This can limit their use in some biomedical applications.

## Challenges

Even when 3D printing offers customizability, design flexibility and multilateral printable option it possesses challenges with modeling to offer a biological purpose [109]. Interaction of the biopolymer with the niche plays a key role in the success of implant. Human body being a system of complex interactions and signaling can be affected due to poor incompatibility arising from foreign body response. The interactions and aspects that needs to be looked upon include immune system and the immunological reactions, systemic effects, integration of implant material with the host tissue, normal mechanical and biochemical interactions, fluid dynamics of bodily fluids and degradation of the material and its metabolism [110]. Multiscale nature is another challenge since the material is encountering wide scale of interactions and thus care should be taken to aptly design the implant. Mechanical, chemical, biological properties together is very crucial for the design and functioning and thus proper understanding is imperative. Limited experimental data in various physiological conditions with respect to recipient customized structural design is also a hindrance.

Biological implants like stents and heart valves needs to be designed conscientiously since it is in constant interaction with non-Newtonian fluid like blood, Therefore the fluid dynamics also needs to be considered apart from the mechanical activity of [111]. Since everyone has unique genome and accordingly the anatomical and physiological characteristics differs. Thus, designing involves understanding of medical image scans, patient details including details of immune reactions and their severity and body fluids [112–115]. All these clinical data need to be critically investigated and integrated before designing using software including Fusion 360 and simplify3D.

The mechanical properties of synthetic polymers used in 3D printing must also be specially designed to meet the requirements of biocompatibility, strength, and durability for usage in biomedical applications. To achieve this, glass or carbon fibers that have been reinforced to increase strength can be used. In order to meet the need for mechanical qualities, it is also possible to ensure strong adhesion by maximizing the extrusion temperature and cooling environment [21, 64]. Controlling infill patterns allows for regulation of porosity, a feature that heavily influences application. The above statement requires the area of 3D printing in biomedical application to be assisted by a material scientist as well to assist in preparing the filament in accordance to the requirement.

Assurance of the implants functioning, and its longevity may not correlate with experimental evidence. Experimental analysis in organisms is also an ethical issue thus in vivo analysis is always not possible. Limited access to human samples along with complexity of recreation of in vivo condition appropriate to human or other animal model hinders the possibility to validate the results. To combat these challenges, a multidisciplinary approach rich in knowledge of engineering, material science, clinical knowledge, biology, and modeling is necessary.

## Conclusion

Polymers have been rapidly researched, especially for their applications in the medical and health sectors, because of their desired properties like biodegradability and biocompatibility. The usage of polymeric substances and synthetically derived materials are proven to make positive changes in the medical sector and make health care effective and cheaper with affordable treatment costs. It is important to consider various factors before utilizing them to prevent any unwanted effects on the body. Biocompatibility, bio inertness, bioactivity or surface reactivity, biodegradability, sterilizability, sufficient mechanical and physical qualities, manufacturability, low weight, cost-friendly, etc. are the main factors among them. Synthetic biopolymeric materials in biomedical applications possess various promising properties, such as mechanical, physical, chemical, thermal, biocompatibility, and optical property. The use of biopolymers like polyethylene, polypropylene, polycaprolactone, polylactide, etc. for biomedical applications varies from one polymer to another based on the characteristics they possess as mentioned above. Synthetic polymers which include polypropylene, polycaprolactone, acrylonitrile butadiene styrene, nylon, polylactic acid, polyether ether ketone, polyether ketone, glycolic acid, polycarbonates, and polyamides have proven biomedical applications longing from drug delivery systems, medical implants, sutures, and catheters, etc. Future developments of different biopolymeric applications for therapeutic systems, incorporating the capacity to target the drug to the drug receptor site. Historically, the biocompatibility of materials has concentrated on minimizing biological interactions to reduce the possibility of non-cellular transplant rejection of pacemakers and other biomaterials. Now, with the help of 3D-printed biomaterials, personalized and compatible approaches can be assured to combat poor biocompatibility issues. Recent research focuses on developing novel 3D printable materials to enhance the biomedical sector. Researchers and medical personnel can produce extremely accurate and customized structures with regulated mechanical properties by mixing synthetic polymers with resin. This enables the creation of individualized implants and prosthetics, improving patient outcomes and enhancing tissue integration. For the benefit of biomedical applications, resin-based 3D printing of biopolymers employing synthetic polymers is a significant technique. Undoubtedly, further study and development in this field will open new doors and result in improvements in healthcare, regenerative medicine, and personalized therapies.

## Future scope

Designing future functional materials for a biomedical application requires a thorough understanding of biopolymer networks. Enhancing their properties expand the range of applications for them in various fields, including medicine. For the medical industry, it is necessary to evolve new biopolymers that will achieve a high level of biocompatibility when the appropriate alterations are made to their chemical and phase composition. Additionally, with more study and comprehension, it will be possible to create fully functional organs in an in vitro culture that can function as designed in a patient’s body using synthetic or natural polymer matrices. This can be achieved by an understanding of a specific organ, its mechanics, and its physiology. Therefore, greater emphasis should be given to 3D printing technology for a variety of tissue engineering applications. With the help of this promising and encouraging technology, we could address the issue of shortage of tissues and organs required for transplants and develop systems to create materials specifically for various types of tissues apart from making implants and biomedical devices with the economic notion. Future uses of resin for biopolymer 3D printing with synthetic polymers in biomedical applications seem hopeful. The potential uses include customized implants, tissue engineering scaffolds, drug delivery systems, bioactive implants, and organ-on-a-chip systems. Furthermore, applications from artificial intelligence and machine learning might assist in design in the future of additive manufacturing [65].

## Abbreviation

PLA: Polylactic acid-PLA,
PVA: Polyvinyl alcohol
PCL: Polycaprolactone
PLGA: Polylactic-co-glycolic acid
PA: Polyamide
PEEK: Polyether ether ketone
PP: Polypropylene
PC: Polycarbonates
PGA: Polyglycolic acid
ABS: Acrylonitrile butadiene styrene
PAEK: Polyaryletherketone
CAPP: Cold atmospheric pressure plasma
ADSCs: Adipose-derived stem cells
hPDLSCs: Human periodontal ligament stem cells
PDL: Natural periodontal ligament
FDM: Fused deposition modeling
SLA: Stereolithography

## References

[1] Van de Velde K, Kiekens P (2002). Biopolymers: an overview of several properties and consequences on their applications. Polym Test.

[2] Niaounakis M. Biopolymers: applications and trends. William Andrew; Elsevier; 2015.

[3] Carlini AS, Adamiak L, Gianneschi NC (2016). Biosynthetic polymers as functional materials. Macromolecules..

[4] Rebelo R, Fernandes M, Fangueiro R (2017). Biopolymers in medical implants: a brief review. Procedia Eng.

[5] Poole-Warren L, Martens P, Green R, editors. Biosynthetic polymers for medical applications. Woodhead publishing series in biomaterials. Elsevier; 2015.

[6] Biswas MC, Jony B, Nandy PK, Chowdhury R, Halder S, Kumar D, Ramakrishna S, Hassan M, Ahsan A, Hoque E, et al. Recent Advancement of Biopolymers and Their Potential Biomedical Applications. J Polym Environ. 2022;30:51–74.

[7] Mtibe A, Motloung MP, Bandyopadhyay J, Ray SS (2021). Synthetic biopolymers and their composites: advantages and limitations—an overview. Macromol Rapid Commun.

[8] Vijay Kumar V, Balaganesan G, Lee JK, Neisiany RE, Surendran S, Ramakrishna S (2019). A review of recent advances in nanoengineered polymer composites. Polymers..

[9] Vijay Kumar V, Ramakrishna S, Kong Yoong JL, Esmaeely Neisiany R, Surendran S, Balaganesan G (2019). Electrospun nanofiber interleaving in fiber reinforced composites—recent trends. Mater Des Process Commun.

[10] Mehmood A, Raina N, Phakeenuya V, Wonganu B, Cheenkachorn K. The current status and market trend of polylactic acid as biopolymer: Awareness and needs for sustainable development. Mater Today Proc. 2022;72:3049–55. 10.1016/j.matpr.2022.08.387.

[11] Subramani PA, Panati K, Lebaka VR, Reddy DD, Narala VR. Nanostructures for curcumin delivery: possibilities and challenges. In: Grumezescu AM, editor. Nano-and microscale drug delivery systems. Elsevier: UK, 2017. p. 393–418.

[12] Heyde M (1998). Ecological considerations on the use and production of biosynthetic and synthetic biodegradable polymers. Polym Degrad Stab.

[13] Tian H, Tang Z, Zhuang X, Chen X, Jing X (2012). Biodegradable synthetic polymers: preparation, functionalization and biomedical application. Prog Polym Sci.

[14] Feldman D, Barbalata A. Synthetic Polymers: Technology, Properties, Applications. Germany: Springer Netherlands; 1996.

[15] Capurro M, Barberis F. 9 - Evaluating the mechanical properties of biomaterials. In: Dubruel P, Vlierberghe SV, editors. Biomaterials for Bone Regeneration. Woodhead Publishing; 2014. p. 270–323, 10.1533/9780857098104.2.270.

[16] Murphy SV, Atala A (2014). 3D bioprinting of tissues and organs. Nat Biotechnol.

[17] Chia HN, Wu BM (2015). Recent advances in 3D printing of biomaterials. J Biol Eng.

[18] Guvendiren M, Molde J, Soares RM, Kohn J (2016). Designing biomaterials for 3D printing. ACS Biomater Sci Eng.

[19] Tappa K, Jammalamadaka U (2018). Novel biomaterials used in medical 3D printing techniques. J Funct Biomater.

[20] Postlethwait RW, Willigan DA, Ulin AW (1975). Human tissue reaction to sutures. Ann Surg.

[21] Zhang P, Gao Q, Yu K, Yao Y, Lu L (2022). Investigation on the temperature control accuracy of a print head for extrusion 3D printing and its improved design. Biomedicines.

[22] Zhang LK, Wang H, Yang R, Liu M, Ban Q, Chen W (2019). Bone marrow stem cells combined with polycaprolactone-polylactic acid-polypropylene amine scaffolds for the treatment of acute liver failure. Chem Eng J.

[23] Pandiyaraj KN, Kumar MR, Kumar AA, Padmanabhan PV, Deshmukh RR, Bah M (2016). Tailoring the surface properties of polypropylene films through cold atmospheric pressure plasma (CAPP) assisted polymerization and immobilization of biomolecules for enhancement of anti-coagulation activity. Appl Surf Sci.

[24] Lim VF, Khoo JK, Wong V, Moore KH (2014). Recent studies of genetic dysfunction in pelvic organ prolapse: the role of collagen defects. Aust NZ J Obstet Gynaecol.

[25] Senarath-Yapa K, McArdle A, Renda A, Longaker MT, Quarto N (2014). Adipose-derived stem cells: a review of signaling networks governing cell fate and regenerative potential in the context of craniofacial and long bone skeletal repair. Int J Mol Sci.

[26] Cheng H, Zhang Y, Zhang B, Cheng J, Wang W, Tang X (2017). Biocompatibility of polypropylene mesh scaffold with adipose-derived stem cells. Exp Ther Med..

[27] Anwar S (2003). The use of prosthetics in hernia repair. Hosp Med.

[28] Becker I, Woodley SJ, Stringer MD (2010). The adult human pubic symphysis: a systematic review. J Anat.

[29] Winnacker M, Rieger B (2016). Poly (ester amide) s: recent insights into synthesis, stability and biomedical applications. Polym Chem.

[30] Elena Gavrila D, Stoian V, Caramitu A, Mitrea S. Advanced Polypropylene and Composites with Polypropylene with Applications in Modern Medicine [Internet]. Composite Materials. IntechOpen; 2021. Available from: 10.5772/intechopen.91783.

[31] Shastri VP (2003). Non-degradable biocompatible polymers in medicine: past, present and future. Curr Pharm Biotechnol.

[32] Sathish T, Palani K, Natrayan L, Merneedi A, De Poures MV, Singaravelu DK. Synthesis and characterization of polypropylene/ramie fiber with hemp fiber and coir fiber natural biopolymer composite for biomedical application. Int J Polym Sci. 2021;2021:2462873.

[33] Rani GU, Sharma S. Biopolymers, bioplastics and biodegradability: an introduction. Encyclopedia of Materials: Plastics and Polymers. 2022;2:474-86. 10.1016/B978-0-12-820352-1.00131-0.

[34] McKeen LW. The effect of long term thermal exposure on plastics and elastomers. William Andrew; 2021.

[35] Dhanasekaran N, Muthuvelu KS, Arumugasamy SK. Recent Advancement in Biomedical Applications of Polycaprolactone and Polycaprolactone-Based Materials. 2022. 10.1016/B978-0-12-820352-1.00217-0.

[36] Singh M, Singh R, Dhami MK. Biocompatible Thermoplastics as Implants/Scaffold. In: Hashmi MSJ, editor. Encyclopedia of Materials: Plastics and Polymers. Oxford, UK: Elsevier; 2022. p. 47–55.

[37] Safi IN, Al-Shammari AM, Ul-Jabbar MA, Hussein BM (2020). Preparing polycaprolactone scaffolds using electrospinning technique for construction of artificial periodontal ligament tissue. J Taibah Univ Med Sci.

[38] Batool F, Morand DN, Thomas L, Bugueno IM, Aragon J, Irusta S (2018). Synthesis of a novel electrospun polycaprolactone scaffold functionalized with ibuprofen for periodontal regeneration: an in vitro andin vivo study. Materials..

[39] Kumar VV, Rajendran S, Balaganesan G, Surendran S, Selvan A, Ramakrishna S (2022). High velocity impact behavior of Hybrid composite under hydrostatic preload. J Compos Mater.

[40] Eshraghi S, Das S (2010). Mechanical and microstructural properties of polycaprolactone scaffolds with one-dimensional, two-dimensional, and three-dimensional orthogonally oriented porous architectures produced by selective laser sintering. Acta Biomater.

[41] Xu S, Li X, Sui G, Du R, Zhang Q, Fu Q (2020). Plasma modification of PU foam for piezoresistive sensor with high sensitivity, mechanical properties and long-term stability. Chem Eng J.

[42] Xu Q, Xu Z, Jiang X, Yarmolenko MA, Rogachev AA, Rogachev AV (2021). Antibacterial coatings based on polycaprolactone and polyurethane with prolonged release of ciprofloxacin. Surf Coat Technol.

[43] Dash TK, Konkimalla VB (2012). Poly-є-caprolactone based formulations for drug delivery and tissue engineering: a review. J Control Release.

[44] Bhattacharjee P, Kundu B, Naskar D, Kim HW, Maiti TK, Bhattacharya D (2017). Silk scaffolds in bone tissue engineering: an overview. Acta Biomater.

[45] Godbole R, Goutam A, Mali A. Microbial Biopolymers: Pharmaceutical, Medical, and Biotechnological Applications. In: Vaishnav A, Choudhary DK, editors. Microbial Polymers. Singapore: Springer; 2021. 10.1007/978-981-16-0045-6_18.

[46] Derakhshanfar S, Mbeleck R, Xu K, Zhang X, Zhong W, Xing M (2018). 3D bioprinting for biomedical devices and tissue engineering: a review of recent trends and advances. Bioact Mater.

[47] Im SH, Im DH, Park SJ, Chung JJ, Jung Y, Kim SH (2021). Stereocomplex polylactide for drug delivery and biomedical applications: a review. Molecules..

[48] Wang Q, Yu X, Chen X, Gao J, Shi D, Shen Y, Tang J, He J, Li A, Yu L, Ding J. ACS Applied Materials & Interfaces. 2022;14(21):24197–24212. 10.1021/acsami.2c05184. 10.1021/acsami.2c05184 35580332

[49] Casalini T, Rossi F, Castrovinci A, Perale G (2019). A perspective on polylactic acid-based polymers use for nanoparticles synthesis and applications. Front Bioeng Biotechnol.

[50] Nahm NJ, Conway JD (2022). Resorbable polylactide membrane for the treatment of segmental bone defects. Injury..

[51] Singhvi MS, Zinjarde SS, Gokhale DV (2019). Polylactic acid: synthesis and biomedical applications. J Appl Microbiol.

[52] Oryan A, Hassanajili S, Sahvieh S (2021). Effectiveness of a biodegradable 3D polylactic acid/poly (ɛ‐caprolactone)/hydroxyapatite scaffold loaded by differentiated osteogenic cells in a critical‐sized radius bone defect in rat. J Tissue Eng Regen Med.

[53] Liang H, Hao Y, Liu S, Zhang H, Li Y, Dong L (2013). Thermal, rheological, and mechanical properties of polylactide/poly (diethylene glycol adipate). Polym Bull.

[54] Norazlina H, Kamal Y (2015). Graphene modifications in polylactic acid nanocomposites: a review. Polym Bull.

[55] Luckachan GE, Pillai CK (2011). Biodegradable polymers—a review on recent trends and emerging perspectives. J Polym Environ.

[56] Mosnáčková K, Opálková Šišková A, Kleinová A, Danko M, Mosnáček J (2020). Properties and degradation of novel fully biodegradable PLA/PHB blends filled with keratin. Int J Mol Sci.

[57] Muthe LP, Pickering K, Gauss C. A Review of 3D/4D Printing of Poly-Lactic Acid Composites with Bio-Derived Reinforcements. Compos. Part C Open Access. 2022;8:100271. 10.1016/j.jcomc.2022.100271.

[58] Guo T, Lim CG, Noshin M, Ringel JP, Fisher JP (2018). 3D printing bioactive PLGA scaffolds using DMSO as a removable solvent. Bioprinting.

[59] Babczyk P, Conzendorf C, Klose J, Schulze M, Harre K, Tobiasch E (2014). Stem cells on biomaterials for synthetic grafts to promote vascular healing. J Clin Med.

[60] Shakiba M, Rezvani Ghomi E, Khosravi F, Jouybar S, Bigham A, Zare M (2021). Nylon—a material introduction and overview for biomedical applications. Polym Adv Technol.

[61] Das D, Mulchandani N, Kumar A, Katiyar V. Fabrication of Stimuli-Responsive Polymers and their Composites: Candidates for Resorbable Sutures. Singapore: Springer; 2020. 10.1007/978-981-15-1251-3_6.

[62] Sullivan MV, Dennison SR, Archontis G, Reddy SM, Hayes JM (2019). Toward rational design of selective molecularly imprinted polymers (MIPs) for proteins: computational and experimental studies of acrylamide based polymers for myoglobin. J Phys Chem B.

[63] Singh S, Prakash C, Ramakrishna S (2019). 3D printing of polyether-ether-ketone for biomedical applications. Eur Polym J.

[64] Tetsuka H, Shin SR (2020). Materials and technical innovations in 3D printing in biomedical applications. J Mater Chem B.

[65] Pugliese R, Regondi S (2022). Artificial intelligence-empowered 3D and 4D printing technologies toward smarter biomedical materials and approaches. Polymers.

[66] Farré-Guasch E, Wolff J, Helder MN, Schulten EA, Forouzanfar T, Klein-Nulend J (2015). Application of additive manufacturing in oral and maxillofacial surgery. J Oral Maxillofac Surg.

[67] Giannatsis J, Dedoussis V (2009). Additive fabrication technologies applied to medicine and health care: a review. Int J Adv Manuf Technol.

[68] Pakhaliuk V, Poliakov A (2018). Simulation of wear in a spherical joint with a polymeric component of the total hip replacement considering activities of daily living. Facta Univ Ser Mech Eng.

[69] Provaggi E, Leong JJ, Kalaskar DM (2017). Applications of 3D printing in the management of severe spinal conditions. Proc Inst Mech Eng Part H J Eng Med.

[70] An J, Teoh JE, Suntornnond R, Chua CK (2015). Design and 3D printing of scaffolds and tissues. Engineering.

[71] Adamzyk C, Kachel P, Hoss M, Gremse F, Modabber A, Hoelzle F (2016). Bone tissue engineering using polyetherketoneketone scaffolds combined with autologous mesenchymal stem cells in a sheep calvarial defect model. J CranioMaxillofac Surg.

[72] Tse I, Jay A, Na I, Murphy S, Niño-Martínez N, Martínez-Castañon GA (2021). Antimicrobial activity of 3D-printed acrylonitrile butadiene styrene (ABS) polymer-coated with silver nanoparticles. Materials..

[73] Okolie O, Stachurek I, Kandasubramanian B, Njuguna J (2020). 3D printing for hip implant applications: a review. Polymers..

[74] Alqurashi H, Khurshid Z, Syed AU, Habib SR, Rokaya D, Zafar MS (2021). Polyetherketoneketone (PEKK): an emerging biomaterial for oral implants and dental prostheses. J Adv Res.

[75] Oladapo B, Zahedi A, Ismail S. Assessing 3D printing of Poly(ether-ether-ketone) and cellular cHAp to increase biointerfaces as a biomedical material. Colloids and Surfaces B: Biointerfaces. 2021;203:111726. 10.1016/j.colsurfb.2021.111726. 10.1016/j.colsurfb.2021.111726 33865088

[76] Vaezi M, Yang S (2015). Extrusion-based additive manufacturing of PEEK for biomedical applications. Virtual Phys Prototyp.

[77] Swider E, Koshkina O, Tel J, Cruz LJ, de Vries IJ, Srinivas M (2018). Customizing poly (lactic-co-glycolic acid) particles for biomedical applications. Acta Biomater.

[78] Teraiya S, Kumar S (2022). Experimental investigation on mechanical properties of additively manufactured anti‐tetrachiral cellular auxetic structures under shear and flexural loading. Polym Eng Sci.

[79] Hashmi AW, Mali HS, Meena A, Saxena KK, Ahmad S, Agrawal MK, Sagbas B, Puerta APV, Khan MI. A comprehensive review on surface post-treatments for freeform surfaces of bio-implants. J Mater Res Technol. 2023;23:4866–908.

[80] Elmowafy EM, Tiboni M, Soliman ME (2019). Biocompatibility, biodegradation and biomedical applications of poly (lactic acid)/poly (lactic-co-glycolic acid) micro and nanoparticles. J Pharm Investig.

[81] Ghosal K, Pal S, Ghosh D, Jana K, Sarkar K. In vivo biocompatible shape memory polyester derived from recycled polycarbonate e-waste for biomedical application. Biomater Adv. 2022;138:212961. 10.1016/j.bioadv.2022.212961 35913244

[82] Jammalamadaka U, Tappa K (2018). Recent advances in biomaterials for 3D printing and tissue engineering. J Funct Biomater.

[83] Liang ZC, Yang C, Ding X, Hedrick JL, Wang W, Yang YY (2021). Carboxylic acid-functionalized polycarbonates as bone cement additives for enhanced and sustained release of antibiotics. J Control Release.

[84] Li M, Ma H, Han F, Zhai D, Zhang B, Sun Y (2021). Microbially catalyzed biomaterials for bone regeneration. Adv Mater.

[85] Guo Z, Liang E, Sui J, Ma M, Yang L, Wang J (2020). Lapatinib-loaded acidity-triggered charge switchable polycarbonate-doxorubicin conjugate micelles for synergistic breast cancer chemotherapy. Acta Biomater.

[86] Kausar A (2018). A review of filled and pristine polycarbonate blends and their applications. J Plast Film Sheeting.

[87] Kumar M, Ramakrishnan R, Omarbekova A (2019). 3D printed polycarbonate reinforced acrylonitrile–butadiene–styrene composites: composition effects on mechanical properties, micro-structure and void formation study. J Mech Sci Technol.

[88] Adewale K. Polyethers. In: Handbook of thermoplastics. United States: CRC Press; 2016. p. 269–302.

[89] Puppi D, Chiellini F (2020). Biodegradable polymers for biomedical additive manufacturing. Appl Mater Today.

[90] Thorat SD, Phillips PJ, Semenov V, Gakh A (2003). Physical properties of aliphatic polycarbonates made from CO2 and epoxides. J Appl Polym Sci.

[91] Koning C, Wildeson J, Parton R, Plum B, Steeman P, Darensbourg DJ (2001). Synthesis and physical characterization of poly (cyclohexane carbonate), synthesized from CO2 and cyclohexene oxide. Polymer..

[92] Liu B, Chen L, Zhang M, Yu A (2002). Degradation and stabilization of poly (propylene carbonate). Macromol Rapid Commun.

[93] Arefin AM, Khatri NR, Kulkarni N, Egan PF (2021). Polymer 3D printing review: materials, process, and design strategies for medical applications. Polymers..

[94] Liu ZL, Zhou Y, Zhuo RX (2003). Synthesis and properties of functional aliphatic polycarbonates. J Polym Sci Part A Polym Chem.

[95] Ucar Y, Akova T, Aysan I (2012). Mechanical properties of polyamide versus different PMMA denture base materials. J Prosthodont Implant Esthet Reconstr Dent.

[96] Cicala G, Latteri A, Del Curto B, Lo Russo A, Recca G, Farè S (2017). Engineering thermoplastics for additive manufacturing: a critical perspective with experimental evidence to support functional applications. J Appl Biomater Funct Mater.

[97] Van De Velde F, De Baets P (1997). The friction and wear behaviour of polyamide 6 sliding against steel at low velocity under very high contact pressures. Wear..

[98] Rahim TN, Abdullah AM, Akil HM, Mohamad D. Comparison of mechanical properties for polyamide 12 composite-based biomaterials fabricated by fused filament fabrication and injection molding. In: AIP conference proceedings, Vol. 1791. AIP Publishing LLC; 2016. p. 020007.

[99] Walker PS, Sathasivam S. Design forms of total knee replacement. Proceedings of the Institution of Mechanical Engineers. Part H, J Eng Med. 2000;214(1);101–19. 10.1243/0954411001535282. 10.1243/0954411001535282 10718055

[100] Moulton SE, Wallace GG (2014). 3-dimensional (3D) fabricated polymer based drug delivery systems. J Control Release.

[101] Yadav P, Yadav H, Shah VG, Shah G, Dhaka G (2015). Biomedical biopolymers, their origin and evolution in biomedical sciences: a systematic review. J Clin Diagnostic Res.

[102] Jaya B, Brajesh B, Fais A, Delogu GL, Kumar A (2022). Biopolymer: a sustainable material for food and medical applications. Polymers..

[103] Ngo TD, Kashani A, Imbalzano G, Nguyen KT, Hui D (2018). Additive manufacturing (3D printing): a review of materials, methods, applications and challenges. Compos Part B Eng.

[104] Pugliese R, Beltrami B, Regondi S, Lunetta C (2021). Polymeric biomaterials for 3D printing in medicine: an overview. Ann 3D Print Med.

[105] Brannigan RP, Dove AP (2017). Synthesis, properties and biomedical applications of hydrolytically degradable materials based on aliphatic polyesters and polycarbonates. Biomater Sci.

[106] Leal‐Egaña A, Scheibel T (2010). Silk‐based materials for biomedical applications. Biotechnol Appl Biochem.

[107] Twaites B, de las Heras Alarcón C, Alexander C. Synthetic polymers as drugs and therapeutics. J Mater Chem. 2005;15:441–55.

[108] Donnaloja F, Jacchetti E, Soncini M, Raimondi MT (2020). Natural and synthetic polymers for bone scaffolds optimization. Polymers..

[109] Tack P, Victor J, Gemmel P, Annemans L (2016). 3D-printing techniques in a medical setting: a systematic literature review. Biomed Eng Online.

[110] Wei F, Liu S, Chen M, Tian G, Zha K, Yang Z (2021). Host response to biomaterials for cartilage tissue engineering: key to remodeling. Front Bioeng Biotechnol.

[111] Wipt P, George KM (2008). 基因的改变NIH public access. Bone..

[112] Krupinski EA (2010). Current perspectives in medical image perception. Atten Percept Psychophys.

[113] Freire-Paspuel B, Vega-Mariño P, Velez A, Castillo P, Gomez-Santos EE, Cruz M (2020). Cotton-tipped plastic swabs for SARS-CoV-2 RT-qPCR diagnosis to prevent supply shortages. Front Cell Infect Microbiol.

[114] Sharan Chandran M, Padmanabhan K (2019). Microbond fibre bundle pullout technique to evaluate the interfacial adhesion of polyethylene and polypropylene self reinforced composites. Appl Adhes Sci.

[115] Mamba’udin A, Handayani M, Triawan F, Rahmayanti YD, Muflikhun MA (2023). Excellent characteristics of environmentally friendly 3D-printed nasopharyngeal swabs for medical sample collection. Polymers..

